# Adsorption of oleic acid on magnetite facets

**DOI:** 10.1038/s42004-022-00741-0

**Published:** 2022-10-23

**Authors:** Marcus Creutzburg, Mine Konuk, Steffen Tober, Simon Chung, Björn Arndt, Heshmat Noei, Robert H. Meißner, Andreas Stierle

**Affiliations:** 1 Centre for X-Ray and Nano Science CXNS, Deutsches Elektronen-Synchrotron DESY, Notkestr. 85, 22607 Hamburg, Germany; 2 Institute of Polymers and Composites, Hamburg University of Technology, Denickestr. 15, 21073 Hamburg, Germany; 3 Department of Physics, University of Hamburg, Luruper Chaussee 149, 22761 Hamburg, Germany; 4 Institute of Surface Science, Helmholtz-Zentrum Hereon, Max-Planck-Str. 1, 21502 Geesthacht, Germany

**Keywords:** Surface spectroscopy, Surfaces, interfaces and thin films, Molecular dynamics, Chemical physics

## Abstract

The microscopic understanding of the atomic structure and interaction at carboxylic acid/oxide interfaces is an important step towards tailoring the mechanical properties of nanocomposite materials assembled from metal oxide nanoparticles functionalized by organic molecules. We have studied the adsorption of oleic acid (C_17_H_33_COOH) on the most prominent magnetite (001) and (111) crystal facets at room temperature using low energy electron diffraction, surface X-ray diffraction and infrared vibrational spectroscopy complemented with molecular dynamics simulations used to infer specific hydrogen bonding motifs between oleic acid and oleate. Our experimental and theoretical results give evidence that oleic acid adsorbs dissociatively on both facets at lower coverages. At higher coverages, the more pronounced molecular adsorption causes hydrogen bond formation between the carboxylic groups, leading to a more upright orientation of the molecules on the (111) facet in conjunction with the formation of a denser layer, as compared to the (001) facet. This is evidenced by the C=O double bond infrared line shape, in depth molecular dynamics bond angle orientation and hydrogen bond analysis, as well as X-ray reflectivity layer electron density profile determination. Such a higher density can explain the higher mechanical strength of nanocomposite materials based on magnetite nanoparticles with larger (111) facets.

## Abstract (web summary)

Metal oxide nanoparticles functionalized by organic molecules are important building blocks for nanocomposites. Here, the authors study the adsorption of oleic acid on magnetite (001) and (111) surfaces experimentally by SXRD, LEED, FT-IRRAS and XRR, and employ a molecular dynamics force field specifically developed for magnetite/organic interfaces to determine molecular adsorption geometries, coverages, layer thicknesses and adsorption-induced structural changes in the substrate.

## Introduction

Metal oxide nanoparticles (NPs) functionalized by organic molecules are important building blocks for novel nanocomposites in materials science with exceptional bending modulus, hardness, and mechanical strength^1–3^. The interaction of organic molecules with the oxide nanoparticle surfaces and the interlinking of the molecules is argued to be the key element for their exceptional mechanical stability. Moreover, magnetite is an efficient material for the removal of glyphosate from water^4^. Employed in biomedical imaging or as drug carriers in medicine^5^, iron oxide nanoparticles also have a promising potential for theranostatic applications, e.g. magnetic hyperthermia^6^. In order for targeted drug delivery or theranostic tissue engineering, nanoparticles must, however, be functionalized. Monodisperse magnetite nanoparticles starting from a few nanometers in size up to 50 nm or more can be formed into various shapes ranging from cubic to truncated cuboctahedrons^7–11^. A common synthesis route for magnetite nanoparticles is precipitation from a precursor in a bath of boiling oleic acid (OLAC), leading to monodisperse nanoparticles with defined, OLAC covered facets^12^. OLAC is a preferred organic ligand for many applications because it can bind strongly to the oxide surface, but it also allows the easy customization of the functionalization by ligand exchange^2^.

During synthesis OLAC stabilized magnetite nanoparticles mainly form (001)-type and (111)-type facets, but not much is known about the ligand arrangement on different surfaces and ligand adsorption-induced faceting mechanisms^1^. The molecular binding geometry, orientation and density, as well as the near-surface structure and composition of the oxide nanoparticles influence the nanoparticle growth, binding, interface structure, and ultimately their performance in the above mentioned applications. Using Fourier transform infrared reflection-absorption spectroscopy (FT-IRRAS), OLAC was found to dissociate on the nanoparticle surface^1^ and adapting chelating adsorption geometries^13^ (both carboxylate oxygen atoms bound to the same metal substrate atom), or bidentate bridging (both carboxylate oxygen atoms bound to two metal substrate atoms) together with non-dissociated molecules^14^, or both mono- and bidentate geometries^15^. However, the connection between binding mode and magnetite surface orientation is still missing. It has also been experimentally observed that the formation of the rod-like morphology of magnetite nanoparticles is a result of the growth along their (110) direction which was attributed to the preferential adsorption of oleate on the (111) facets of Fe_3_O_4_^16^. A microscopic explanation of this observation is again lacking.

Formic acid, the simplest carboxylic acid, is an important prototypical molecule for the adsorption of larger carboxylic acids like OLAC, and exhibits the same carboxylic end group which binds to the oxide surface. On the magnetite (001) surface, formic acid adsorbs in a bidentate bridging geometry at room temperature with a saturation coverage of 2.8 molecules/nm^2^^17,18^. The magnetite (001) surface presents a $(\sqrt{2}\times \sqrt{2})$ R 45° subsurface cation vacancy (SCV) reconstruction after preparation under ultra-high vacuum (UHV) conditions^19,20^. The reconstruction of this oxygen rich surface, consisting of two subsurface octahedral (oct) vacancies and one additional tetrahedral (tet) iron ion, is lifted upon adsorption of atomic hydrogen, water^21^, or formic acid^17,18^ by iron cation rearrangement at room temperature, reestablishing a stoichiometric surface.

The surface termination of magnetite (111) is preparation-dependent and multiple terminations can coexist^22^ altering the adsorption behavior. The magnetite (111) single crystal surface is terminated by tetrahedrally coordinated iron ions (layer notation Fe_tet1_) over a closed packed oxygen layer after (reducing) UHV preparation. The Fe_tet1_ layer presents around 20% point defects (Fe_tet_ vacancies) making oxygen-terminated areas accessible^23^. Formic acid adsorbs on this surface in two different geometries: chelating and quasi-bidentate i.e., one carboxylate oxygen atom bound to a Fe_tet1_ ion and the other bound to an OH group on the surface. The saturation coverage of formic acid is higher than on magnetite (001) and reaches up to 3.3 molecules/nm^2^.

Understanding the carboxylic acid/oxide interface starting from adsorption on planar (001)- and (111)-type facets of magnetite, the most prominent crystal orientations on magnetite NPs, is of utmost importance for many fields in materials science, such as the development of new nanocomposites with tailored mechanical properties. In the present work, we studied the adsorption of OLAC on magnetite single-crystalline (001) and (111) facets under UHV conditions focussing on adsorption coverage and binding geometries as well as the structural changes in the substrate using complementary FT-IRRAS, low energy electron diffraction (LEED), X-ray reflectivity (XRR), surface X-ray diffraction (SXRD) and molecular dynamics (MD) simulations. Investigating the adsorption under (reducing) UHV conditions is comparable with the conditions used to produce magnetite nanoparticles from a FeO(OH) precursor^1^. Although, it should be noted, that we employ specific conditions to ensure clean oxide surfaces rather than the usual thermal decomposition reaction found in nanoparticle synthesis^12^. While an ab initio-based description of the interface between OLAC and magnetite would be advantageous due to the reactivity of OLAC, the vast configurational freedom and intricate adsorption structures that OLAC exhibits on both surfaces prevent the use of such approaches, especially when studying high surface coverages. However, a properly parameterized force field provides an effective and accurate remedial strategy.

Our MD simulations, optimized specifically for the accurate description of dissociative adsorption of carboxylic acids^24^, allow a rigorous determination of the interfacial properties and, by comparison with experimental results, illustrate the transferability of our empirical force field to complex carboxylic acids on magnetite surfaces.

## Results and discussion

### Oleic acid adsorption-induced structural surface changes

**Fig. 1:**
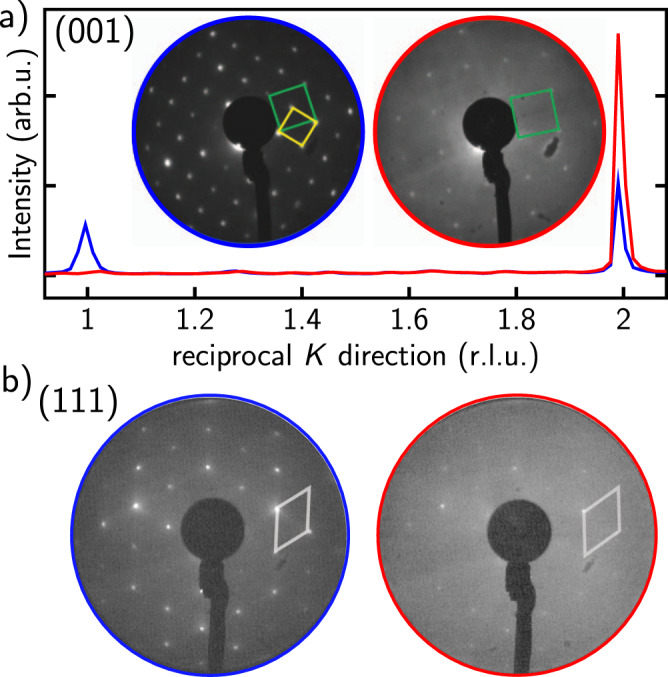
LEED patterns before and after the adsorption of OLAC. **a** Line scans along reciprocal *K* direction to monitor the SCV reconstruction and the CTR signal on magnetite (001) before (blue) and after OLAC adsorption (red). Inset: LEED patterns (electron energy 68 eV) show the SCV reconstructed surface (blue circle) and after OLAC adsorption (red circle). **b** LEED patterns of the clean magnetite (111) surface obtained at 125 eV (blue circle) and after OLAC adsorption (red circle). The reciprocal unit cell is indicated by a white rhombus.

**Fig. 2:**
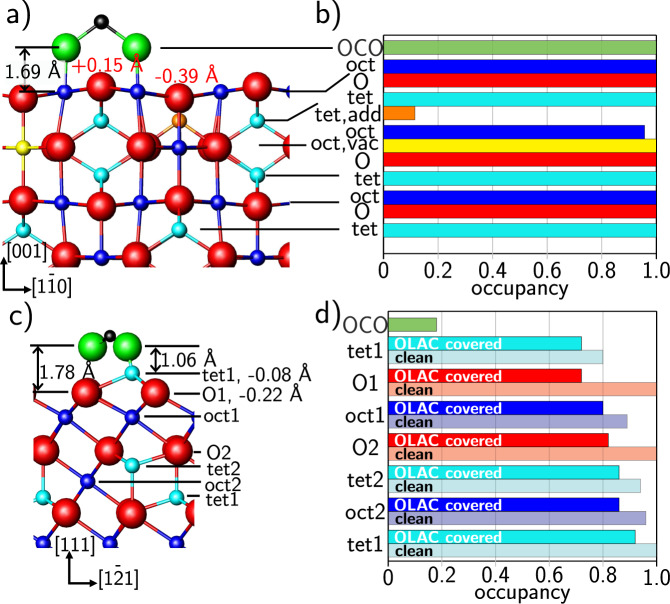
Structural changes upon OLAC adsorption. Structural model obtained from SXRD fit of magnetite (001) (**a**) and (111) (**c**) with atomic displacements with respect to the bulk positions. Figures (**b**) and (**d**) show the occupancy profiles obtained from the SXRD fits which correspond to the respective atomic layer of the unit cell in (**a**) and (**c**) for magnetite (001) and (111), respectively. Magnetite oxygen is shown in red, carboxylate oxygen in green, carbon in black, octahedral iron in blue, tetrahedral iron in light blue, octahedral vacancy sites in yellow and tetrahedral interstitial ions in orange.

The structural response of the magnetite (001) and (111) surfaces upon OLAC adsorption was first investigated by LEED and SXRD, see Figs. 1 and 2. The clean surfaces were exposed to OLAC for 60 min at 1 × 10^−6^ mbar and room temperature. The LEED patterns of the (001) surface before and after OLAC adsorption are shown in the inset of Fig. 1a. The clean (001) surface exhibits a $(\sqrt{2}\times \sqrt{2})$ R45° reconstruction (reciprocal lattice unit cell: yellow square), the green square indicates the unit cell of the unreconstructed surface^19^. After OLAC adsorption, the superstructure spots of the SCV reconstruction disappeared, indicating a lifting of the reconstruction, which was also observed for formic acid adsorption on the magnetite (001) surface^17,18^. The LEED pattern was also attenuated and got diffuse, which indicates that the adsorbed OLAC molecules are disordered without any preferential orientation of their aliphatic tail.

We obtained quantitative information on the OLAC adsorption-induced near-surface structural changes from SXRD. A line scan through reciprocal space at *H* = 2 and *L* = 1.6 along the *K* direction is shown in Fig. 1a. It probes both a surface reconstruction signal at *K* = 1 and a crystal truncation rod (CTR) signal at *K* = 2^20^. The surface reconstruction signal vanished after OLAC adsorption and the CTR signal was enhanced, characteristic for reconstruction lifting and a strong near-surface structural rearrangement and cation diffusion from the bulk at room temperature, as it was also observed after formic acid^17^ or atomic hydrogen^21^ adsorption. The results of the quantitative analysis of the SXRD data (Fig. S4 of the SI, together with fit) are shown in Fig. 2a, b. For the fit a bulk-truncated surface model with one deprotonated carboxylic group per surface unit cell was employed. The carboxylate group was placed on top of two adjacent octahedral iron ions in bidentate bridging geometry (in line with our FT-IRRAS results, see below) and centered at the subsurface tetrahedral iron sites. This adsorption site was preferred for the adsorption of formic acid on magnetite (001)^17^. Due to the high degree of freedom and consequently a high Debye–Waller factor the aliphatic chain of the OLAC molecule was not included in the fit. The *z* displacements (along the surface normal) in the topmost iron layer show a smaller relaxation (−0.011 Å) compared to the clean surface (−0.296 Å). This is expected since there is an adsorbate on top and it is also consistent with the adsorption of formic acid on the magnetite (001) surface^17^. Displacement (and occupancy) error bars are given in Supplementary Discussion 1. For the OLAC-covered surface the out-of-plane distance between carboxylate oxygen and the top layer magnetite oxygen is 1.69 Å and thus closer than for formic acid on this surface (2.00 Å). From MD simulations a distance of 1.92 Å is obtained, which is in good agreement to the SXRD fit considering the use of an empirical force field. Oxygen ions in the subsurface layer displace (in *z*) towards the octahedral iron ions which is consistent with the formic acid covered surface^17,25^. The iron *z* displacements into the bulk in the subsurface layers are consistently higher for OLAC than for formic acid which is due to the stronger interaction of OLAC with the surface, compare Table S1.

The occupation profile of the different atomic layers obtained from the fit is shown in Fig. 2b (the fitted atomic positions and occupancies of the clean (001) surface and after OLAC adsorption are given in Tables S2 and S3, respectively). We find that the two subsurface octahedral vacancies of the clean, reconstructed surface are nearly completely filled after adsorption and the other adjacent octahedral iron sites are occupied by 96%. The subsurface interstitial tetrahedral iron ion site remains filled to 11%. Therefore, we conclude that the surface reconstruction is almost completely lifted. The lifting mechanism follows the same path as for the adsorption of formic acid, water vapor or atomic hydrogen on this surface^17,21^. In all these cases, atomic hydrogen plays a key role, which indicates that in the case of OLAC adsorption the carboxylic end group dissociates and the released H induces the near-surface cation redistribution. As a result, the bulk stoichiometry of the surface is almost reestablished during the OLAC adsorption process (the SCV reconstructed surface is oxygen rich), without further vacancy formation at the interface. Such a vacancy formation was observed in contrast to the interaction of water vapor and atomic hydrogen with the (001) surface.

In the following, we will compare with OLAC adsorption-induced structural changes of the magnetite (111) surface. The LEED pattern of the clean (111) surface, terminated by tetrahedrally coordinated Fe ions^26^ is shown in Fig. 1b. The surface is unreconstructed and upon OLAC adsorption, the LEED signal is more strongly attenuated as compared to the (001) surface, indicating the formation of a thicker organic layer. At the same time, the diffuse background increased and no additional diffraction spots became visible, inline with disordered adsorption already observed for the (001) surface. The quantitative analysis of the CTR data (Figs. S5, 2c, d, Table S5 and Supplementary Discussion 2) confirms for the clean surface a tetrahedral Fe_tet1_ surface termination^23^, together with 20% Fe vacancies in the topmost layer. For the fit of the OLAC exposed surface, a carboxylic end group was placed in quasi-bidentate geometry. The structure factor difference is negligible when it is placed into the chelating site.

The *z*-displacement of the Fe_tet1_ surface layer is reduced after adsorption to −0.079 Å (clean surface: −0.134 Å). A similar trend of the topmost Fe and O ions showing smaller relaxations after adsorption was also observed for OLAC on magnetite (001) and indicates that the surface can now compensate its polarity by charged adsorbates instead of purely relaxations in the case of the clean surface. After adsorption of OLAC (1 × 10^−6^ mbar, 60 min at room temperature), we find lower subsurface iron and oxygen occupancies of the first four layers compared to the clean surface, see Fig. 2d. We interpret this as an adsorption-induced vacancy formation. In this scenario, atomic hydrogen adsorbs on surface oxygen forming OH and subsequently recombines with another H forming water. Water molecules then desorb from the surface while iron ions diffuse from the bulk to interstitial sites. A similar vacancy formation was reported for the adsorption of hydrogen^21^ on magnetite (001) and formic acid on magnetite (111)^27^. It might be beneficial for the OLAC layer growth since more active sites, edge and corner atoms, are available at the surface. Thus our experiments show that as a result of the vacancy formation, additional iron and oxygen vacancies are formed on magnetite (111) after adsorption. This is in contrast to the filling of vacancies (lifting of the SCV reconstruction) after the adsorption on magnetite (001)^17^. The fitted atomic positions and occupancies of the clean (111) surface and after OLAC adsorption are given in Tables S6 and S7, respectively).

**Fig. 3:**
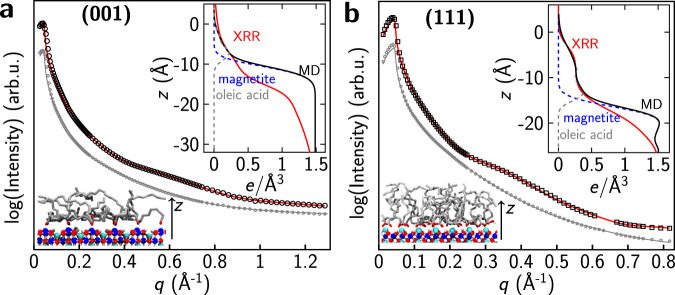
OLAC layer properties investigated with XRR. XRR curves obtained on (**a**) magnetite (001) and (**b**) magnetite (111) before and after OLAC dosing (10^−6^ mbar, 60 min). The gray data points and solid lines depict the respective clean surface XRR data and fit, while data points and fit of the OLAC covered surfaces are shown in black and red, respectively. Curves shifted vertically for clarification. Insets: Electron density profiles as a function of the distance perpendicular to the surface *z*. Experimentally calculated from the XRR fit after OLAC (red line) and estimated for individual components, i.e., magnetite (dashed blue), OLAC (dashed gray), and total (solid black), from MD simulations using the Density Profile Tool as discussed in the main text.

Morphological information on the adsorbed OLAC layers can be obtained by X-ray reflectivity (XRR) independent of their structural order. Figure 3 shows XRR results before and after OLAC adsorption (1 × 10^−6^ mbar, 60 min at room temperature) on the (001) and (111) magnetite surfaces. After OLAC adsorption, there is for both surfaces a distinct oscillation visible in the XRR curves, which results from constructive and destructive interference due to the electron density difference of the adsorbate layer and the substrate. The oscillation is more pronounced for the (111) surface and shifted to lower momentum transfer *q* values, indicating the formation of a thicker OLAC layer. On the (001) surface, the layer was fitted to a thickness of 8.2 ± 0.8 Å with an electron density of 0.15 ± 0.02 *e*/Å^3^. This corresponds to 0.76 molecules/nm^2^. Our previous studies^17,18^ show that formic acid adsorbs with two molecules per unit cell on magnetite (001) (2.8 molecules/nm^2^). A higher molecule density can be achieved for formic acid because of the smaller molecule size. In addition, the orientation of the aliphatic chain of OLAC has to be taken into account as it is effectively blocking adsorption sites on magnetite (001), see FT-IR and MD sections below. The XRR fit parameters are given in Table S4. The insets in Fig. 3 show the electron density along the surface normal calculated from the XRR fit and as a comparison, the electron density profiles calculated from MD simulations for the same coverage (details see MD paragraph).

On the (111) surface, the OLAC layer thickness was determined to be 14.1 ± 1.4 Å with an electron density of 0.26 ± 0.03 *e*/Å^3^, see inset in Fig. 3b and XRR fit parameters in Table S8, which is still below the bulk OLAC electron density at room temperature of 0.37 *e*/Å^3^. The fitted density corresponds to 2.33 OLAC molecules/nm^2^. The fitted layer thickness suggests a more upright orientation of the molecules (length: 20 Å) on the (111) surface as compared to the (001) surface. The electron density and thickness of the OLAC layer are higher on the (111) surface suggesting a different adsorption behavior than on the (001) surface. A surface coverage of 1.9 OLAC molecules/nm^2^ was reported on magnetite nanoparticles after a thermal treatment at 150 °C^1^, in good agreement with our results, considering a mixture of (001) and (111)-type facets in the nanocomposite material. Unfortunately, no additional information of the surface morphology could be obtained from STM, see Supplementary Notes 4 and Figs. S6 and S7.

### Molecular adsorption geometry determined by FT-IRRAS

**Fig. 4:**
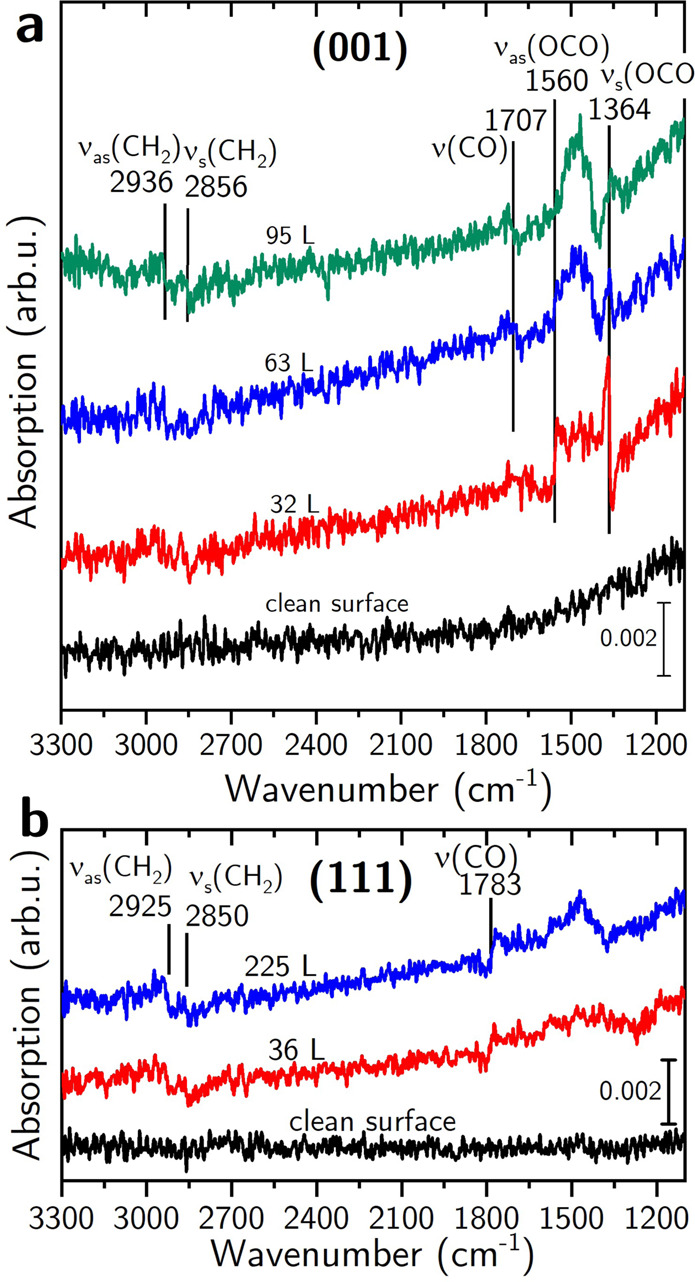
FT-IRRAS of adsorbed OLAC on magnetite. p-polarized FT-IRRA spectra of OLAC on the two magnetite surfaces. OLAC exposure on magnetite (001) (a) and (111) (b) was done at 7 ⋅ 10^−8^ and 2 ⋅ 10^−7^ mbar, respectively. See main text for more information.

FT-IRRAS measurements were performed on both surfaces to elucidate the binding geometry of OLAC. The *p*-polarized spectra acquired after OLAC adsorption on magnetite (001) are shown in Fig. 4a. Upon adsorption two vibrational bands appear at low coverage at 1560 cm^−1^ and 1364 cm^−1^. Both bands exhibit a Fano-type line shape, which results from magnetite being neither a perfect metal nor a perfect insulator^17,18,28^. The band at 1364 cm^−1^ presents a line shape which is expected for a dynamical dipole perpendicular to the surface^17,23^. The one at 1560 cm^−1^ has an inverted line shape which originates from a dynamical dipole parallel to the surface. Both bands appear at similar positions as observed after 2 L (1 Langmuir = 1.33 × 10^−6^ mbar × s) formic acid exposure on magnetite (001) (1544 and 1368 cm^−1^^17,18^). On magnetite (001) they were assigned to the asymmetric and symmetric stretching bands *ν*_as_ and *ν*_s_(OCO) of formate in a bidentate bridging adsorption geometry. OLAC stabilized magnetite nanoparticles show vibrational bands at 1603 cm^−1^ and 1377 cm^−1^ which were assigned to the same stretching modes^1^. Hence, we conclude that for low coverage OLAC dissociates to oleate (C_17_H_33_COO^−^) and binds with its carboxylic end group to the magnetite (001) surface in the same bidentate bridging configuration as formic acid does: with two oleate oxygen atoms bound to two octahedral iron ions on the surface. The larger splitting between *ν*_as_ and *ν*_s_ in the case of OLAC (196 cm^−1^) compared to formic acid (176 cm^−1^) is, however, not reflected in a larger OCO bond angle of oleate (117.5° from MD simulations, see Table S9) compared to formate (125.5° from DFT^17^). This trend was seen for formate in different adsorption geometries on the Fe_3_O_4_(111) surface^23^, indicating that also the aliphatic tail has an influence on the splitting. With increasing OLAC coverage the two *ν*_s_(OCO) bands became broader, overlap and shift, likely due to increasing disorder and intermolecular interaction. An additional band at 1707 cm^−1^ appears for higher exposure and is assigned to the C=O stretching vibration of non-dissociated OLAC molecules^13,18^. The shape of this band is the same as for *ν*_s_(OCO), hence resulting from a net dynamical dipole perpendicular to the surface. Along with the initial dissociation of OLAC on magnetite (001) we observe pronounced adsorption of non-dissociated molecules with the C=O bond in a preferred orientation (for more details, see MD section). Two additional bands at 2936 cm^−1^ and 2856 cm^−1^ appear at higher exposure and are assigned to *ν*_as_ and *ν*_s_ stretching vibrations of CH_2_.

The FT-IRRAS results after OLAC exposure of the clean (111) surface are shown in Fig. 4b. After dosing at a pressure of 2 × 10^-7^ mbar for 4 min a broad contribution with its maximum centered around 1470 cm^−1^ appears, which further grows at higher exposures. This broad band can also be observed in the high coverage spectra of OLAC on the (001) surface, where it was assigned to an overlap of the *ν*_s_(OCO) and *ν*_as_(OCO) of the carboxylic end group. We therefore suggest that the OLAC carboxylic end group also dissociates on the magnetite (111) surface at room temperature and that the *ν*_as_(OCO) and *ν*_s_(OCO) overlap due to higher intermolecular interaction. The dissociation of formic acid was observed as well on magnetite (111) and formate in quasi-bidentate and chelating adsorption geometries, giving rise to three pronounced bands in the *ν*(OCO) stretching region^23^. Another band appears at 1787 cm^−1^ after an exposure to 2 × 10^−7^ mbar for 4 min and is attributed to the *ν*(C=O) stretching vibration of non-dissociated OLAC molecules. The shape of this band matches the line shape that is expected for a dynamical dipole parallel to the surface which is in direct contrast to the perpendicular orientation on magnetite (001). Similar to the (001) surface, the theoretical OCO bond angle is smaller for oleate compared to formate in identical adsorption geometries (chelating: 112.4° for oleate (MD), compared to 120° for formate (DFT); quasi-bidentate: 115.5° for oleate, compared to 128° for formate, see Table S9 and ref. ^23^). The smaller OCO bond angles for oleate are likely due to the larger molecule size and increasing molecule disorder at higher coverages. Two bands with a small amplitude appear around 2923 cm^−1^ and 2850 cm^−1^. These bands are assigned to the asymmetric and symmetric *ν*(CH_2_) stretching vibrations, respectively, similar to the (001) surface. The band position in both cases is slightly reduced compared to the free molecule^13^ due to the limited mobility of the alkyl chains.

### Molecular dynamics simulation of oleic acid adsorption

**Fig. 5:**
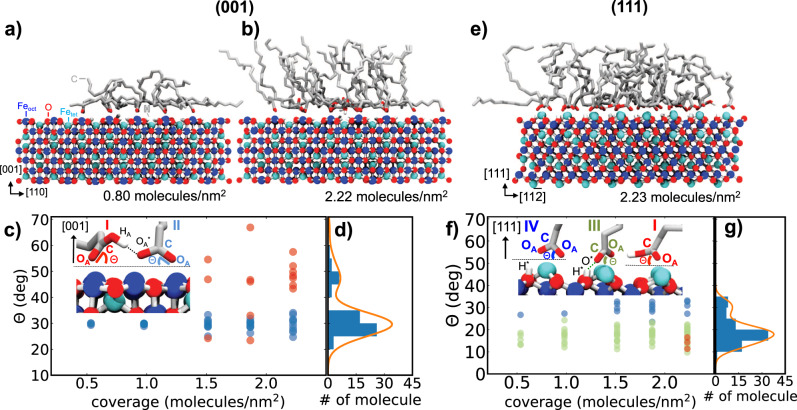
Ligand structure of OLAC on magnetite. Ligand structure on magnetite (001) (**a**, **b**) and on (111) (**e**) at OLAC/oleate coverages indicated below the structures. Exemplary adsorption configurations of OLAC/oleate on magnetite, i.e. molecular (I), bidentate (II), quasi-bidentate (III) and chelating (IV), are shown in the insets of (**c**) and (**f**) and are explained in the main text. Different colors are used in (**c**) and (**f**) to indicate angles Θ between C–O_A_ (C = O_A_ in the case of OLAC) and the surface plane. Colors refer to the structures (I–IV) given in the inset. Histograms in (**d**) and (**g**) show the distribution of Θ for the highest coverage on both surfaces.

At early stages of the ligand adsorption, molecule-molecule interactions play only a minor role and OLAC finds an adsorption site on both surfaces almost immediately upon iterative deposition. Every new attempt to add OLAC to the system leads thus to a dissociative adsorption of OLAC to oleate (structure II, III, or IV in the insets of Fig. 5). A summary of the different adsorption geometries is given in Supplementary Discussion 3 and Fig. S8. Associated structural features are summarized in Table S9. Consequently, the dissociative adsorption of OLAC up to a coverage of around 1.07 molecules/nm^2^ quickly forms a monolayer on the (001) surface which is in good agreement with FT-IRRAS results.

Above this coverage, the time required for newly introduced OLAC to find an adsorption site where it can dissociatively adsorb to oleate increases dramatically and molecular adsorption (structure I in the inset of Fig. 5c) starts to occur. Angles, denoted by Θ, formed between the C=O_A_ bond of OLAC (red dots) or the C–O_A_ bond of oleates (blue dots) and the (001) surface at different coverages are shown in Fig. 5c. In the early stages of layer growth on the (001) surface, Θ is found to be around 30°. Above a coverage of 1.07  OLAC/nm^2^ the distribution of Θ on (001) starts to shift to larger angles in which molecularly adsorbed OLAC binds to a Fe_oct_ via the oxygen of the C=O_A_ group while establishing a hydrogen bond to another oleate already adsorbed on the surface (structure I in the inset of Fig. 5c). The C=O_A_ bond of the molecularly adsorbed OLAC typically adopts a more upright configuration with angles Θ of 45° ± 25°. This finding is in line with our FT-IRRAS results where the dynamical dipole moment of the *ν*(C=O) band at 1707 cm^−1^ indicates that the C=O bond is oriented more perpendicular to the surface for higher ligand coverages. In general, we expect that simulations comprising a larger surface area are more sensitive to the detection of molecular adsorption of OLAC and that it will start even earlier. The hydrogen bond formed between a molecularly adsorbed OLAC and oleate (1.57 Å) is significantly shorter than hydrogen bonds formed between molecularly adsorbed OLAC and surface oxygens (2.53 Å). Up to a coverage of 1.50 molecules/nm^2^, the aliphatic chains lie rather flat on the (001) surface and are disordered (cf. Fig. S11), effectively blocking adsorption sites. At surface coverages of 2.22 molecules/nm^2^ (Fig. 5b), a more vertical alignment of the aliphatic chains with respect to the surface is observed, eventually leading to a self-assembled layer when the surface coverage further increases. It is instructive to compare the formation energies for the monolayer buildup as was done previously in Dietrich et al.^29^. We provide the associated formation energies calculated using this recipe, which corrects the force field energy for the correct quantum chemical energy of the proton transfer reaction, compare Fig. S9. It is interesting to note that the energy difference between the adsorption of formic acid and oleic acid, using this correction, is about 0.5−0.7 eV, which is similar to the results of Dietrich et al.^29^ where 1.2 eV was determined for the aliphatic chain contribution. Energetically, we first see a sharp drop associated with the dissociative adsorption of oleic acid on both surfaces. In the case of magnetite (111) two different slopes appear which we attribute to the change from a quasi-bidentate to a chelating adsorption. Thereafter, the geometric criterion of dissociation is no longer satisfied, and we observe molecular adsorption of oleic acid forming a hydrogen bond to a nearby oleate. We stopped adding new oleic acids when we could no longer observe this molecular adsorption.

Electron density profiles were calculated for the experimentally determined coverage of 0.8 molecules/nm^2^ on the (001) surface and are shown in the inset of Fig. 3a. The layer thickness is around 8.33 ± 0.85 Å in our simulations which is in very good agreement with the XRR determined thickness of 8.2 Å. Since the adsorption is only partially dissociative and not all adsorption sites are occupied, as it is the case for formic acid, the surface is also only partially hydroxylated—a possible explanation why the reconstruction is not completely lifted. A visualization of the iterative dissociative adsorption of OLAC on magnetite (001) is shown in Supplementary Movie 1 and Fig. S12.

Depending on the coverage, also for the (111) surface different adsorption motifs of molecularly adsorbed OLAC and oleates are observed (cf. Fig. 5f). A higher tendency for a quasi-bidentate binding is observed, in which one of the oleate oxygens (O_A_) binds to a Fe_tet1_ and the other oxygen binds via a hydrogen bond to the closest surface hydroxyl (structure III in the inset of Fig. 5f). Only at higher surface coverages, a chelating configuration is observed more frequently (structure IV). Molecular adsorption on magnetite (111) is only observed at very high surface coverages above 2.23 OLAC molecules/nm^2^ (structure I in the inset of Fig. 5f) in which OLAC binds via the oxygen of the carbonyl group to a Fe_tet_ atom and (preferentially) via a hydrogen bond to another adsorbed quasi-bidentate oleate. The angles of the different adsorbed OLAC and oleate groups behave as Θ_chelate_ > Θ_quasi-bidendate_ ≥ Θ_molecular_ and an angle of 15° was found for the C=O bond of molecularly adsorbed OLAC (red dots in Fig. 5f). In contrast to the 45° orientation of the C=O bond of molecularly adsorbed OLAC on the (001) surface, on magnetite (111) the C=O bond has an almost parallel orientation to the surface which is in agreement with the alignment of the dynamical dipole moments found in our FT-IRRAS. Furthermore, aliphatic tails of OLAC at coverages observed experimentally on the (111) surface are in a more upright configuration resulting in a higher layer thickness (see Figs. 5e, S10, S13 and Supplementary Movie 2). The flexibility of the quasi-bidentate binding of oleate on magnetite (111) (hydrogen bonds between formate and surface hydroxyl groups can be easily exchanged, resulting in high rotational flexibility of formate around a Fe_tet1_^24^) leads to less steric hindering of the carboxylic groups adsorbed on the surface (Fig. S10). Consequently, this results in a higher electron density of the layer. In Fig. 3b, the electron density profile perpendicular to the surface is shown and the thickness of the layer with a coverage of 2.23 molecules/nm^2^ was calculated from MD to be 14.42 ± 1.26 Å which is again in very good agreement with the experimentally determined thickness of 14.1 Å via XRR. Observed electron density differences close to the magnetite surface between XRR and MD simulations are attributed to the surface morphology of the experimentally prepared crystal. By combining our experimental results and MD simulation we were for the first time able to determine the adsorption geometries and layer thicknesses and densities of highly relevant OLAC adsorption on magnetite (001) and (111). The determination of dissociative and molecular adsorption for certain OLAC coverages as well as molecule orientation on the two different magnetite surfaces show a high level of agreement between the MD simulations and experimental results making this approach very useful to study the organic acid/oxide interface on the atomic scale.

## Conclusions

In summary, we studied the adsorption of OLAC on the single-crystalline magnetite (001) and (111) facets using FT-IRRAS, LEED, SXRD, and XRR in combination with MD simulations employing a new force field specifically developed for magnetite/organic interfaces. The interfacial properties of OLAC on both magnetite surfaces have distinct differences regarding molecule coverage, layer thickness, binding geometry, and interfacial defects: at low coverage, OLAC dissociates upon adsorption on the magnetite (001) surface at room temperature and adsorbs in a bidentate bridging geometry, just like formic acid does on this surface (underscoring the importance of formic acid as a prototypical molecule for larger carboxylic acids). The bulk stoichiometry is restored after adsorption of OLAC on a (001) surface and for a higher OLAC coverage, non-dissociated molecules adsorb at the surface with the C=O bond oriented perpendicular to the surface and a flat adsorption of the aliphatic OLAC tail, effectively blocking adsorption sites. On magnetite (111), again both dissociative and molecular adsorption of OLAC are observed, however, the latter has a C=O bond parallel to the surface which results in higher thickness and molecular density of the OLAC layer. As previously observed on magnetite (111)^24^, formate molecules exhibit a high mobility at finite temperatures, which can also be a reason for the easier reorganization of the OLAC layer on this surface and thus higher coverages than on the (001) surface. The layer growth of OLAC is much easier on the (111) surface, because proton transfer reaction rates are higher due to the quasi-bidentate and chelating adsorption of oleate leaving enough possible adsorption sites for further agglomeration of FeO(OH) precursor molecules in actual OLAC stabilized magnetite NP composites. This is in line with the annealing behavior in Dreyer et al.^1^, where more (111)-type facets were formed, increasing mechanical strength of the nanocomposite material. The mechanical properties of such composites can be tailored by nanoparticle shape change towards more (111)-type facets and avoiding (001)-type facets since a higher molecule density can be achieved on the former. An accurate atomistic understanding of the interface between magnetite and organic acids is a prerequisite for future upcoming simulations which are able to predict the mechanical properties of nanocomposite bulk materials. While it should be noted that the force field used is capable of accurately describing the stability of common surface terminations on magnetite and even accurately describing the interface with small organic molecules^24^, it should be noted that the simple force field used here cannot incorporate explicit quantum mechanical effects such as magnetism or the specific conductivity of magnetite and provides only a limited representation of the complex charge ordering in magnetite^8^. Nevertheless, our combined microscopic, experimental, and theoretical view of the magnetite/organic acid interface opens up the possibility to tune the mechanical properties of nanocomposite materials and brings this within reach thanks to the availability of reliable force fields and fundamental experiments elucidating this complex interfacial interplay.

## Methods

The natural magnetite (001) single crystal (10 × 10 × 2 mm^3^) was prepared with multiple cycles of Ar^+^ sputtering (5 × 10^−6^ mbar, 1 keV) followed by UHV annealing at 650 °C both for 15 min. The last annealing cycle was done in 1 × 10^−6^ mbar oxygen at 650 °C. Magnetite (111) was prepared by multiple cycles of Ar^+^ ion sputtering (*p*_Ar_ = 5 × 10^−6^ mbar, 0.8 keV) for 10 min, followed by annealing at 700 °C for 10 min in an oxygen partial pressure of ${p}_{{{{\mbox{O}}}}_{2}}=1\times 1{0}^{-6}$ mbar. The substrate was then kept at 700 °C for 5 min at UHV and cooled down to room temperature. Both surfaces were checked with LEED and Auger electron spectroscopy (cf. Supplementary Methods and Fig. S3) prior to OLAC adsorption.

OLAC was dosed at room temperature through a customized UHV nozzle close to the surface at a pressure of around 1 × 10^−6^ mbar. Detailed information about the OLAC dosing system is given in the Supplementary Methods section (Figs. S1, S2).

The FT-IRRAS measurements were carried out in a UHV chamber connected to an FT-IR spectrometer. Each IR spectrum was taken at grazing incidence (~80°) with 512 scans with a resolution of 2 cm^−1^ at a base pressure of 8 × 10^−10^ mbar. Before acquiring each spectrum, residual OLAC in the chamber was pumped out. The measurements were performed at the DESY NanoLab^30^.

The SXRD and XRR data were acquired at the ID03 beamline of the European Synchrotron Radiation Facility (ESRF)^31^ using a 2D detector in stationary mode^32^ and a photon energy of 14 keV. The base pressure was 2 × 10^−10^ mbar. The data integration was done using BINoculars^33^. The data was then fitted to several structural models using ROD from the ANAROD package^34^. The SXRD data presented here are referenced according to the cubic surface unit cell of magnetite (001) described by the following parameters: *a* = *b* = *c* = 8.394 Å along the [1,0,0], [0,1,0] and [0,0,1] bulk directions, respectively, using *α* = *β* = *γ* = 90°. The magnetite (111) SXRD data are referenced to the hexagonal surface unit cell with the following parameters: *a* = *b* = 5.935 Å, *c* = 14.539 Å along the [1,$\overline{1}$,0], [0,1,$\overline{1}$] and [1,1,1] bulk directions, respectively, using *α* = *β* = 90° and *γ* = 120°. Additional information is given in the Supplementary Notes 1. The XRR data are plotted as a function of the momentum transfer along the surface normal: $q=4\pi {\lambda }^{-1}\sin \theta$ with *λ* = 0.8856 Å (*E*_*γ*_ = 14 keV) and the incidence angle *θ*. The electron density profiles, which are calculated from the fit, are plotted in *e*/Å^3^ as a function of the distance perpendicular to the surface *z*, as shown in the insets of Fig. 3. Fitting was done with the Fewlay program^30^ using a three-layer model consisting of OLAC, an intermediate magnetite layer, and a bulk magnetite layer. The intermediate layer has a reduced electron density compared to bulk magnetite and can be explained by island growth during annealing^35^. The XRR fit parameters can be found in Supplementary Notes 2 and 3, Tables S4 and S8, respectively.

All MD simulations were performed using LAMMPS^36^ utilizing a Nosé-Hoover thermostat (NVT)^37,38^. Equations of motion are integrated with timesteps of 0.5 fs in the velocity-Verlet algorithm. A standard Ewald summation^39^ with a precision of 10^−6^ and a cutoff of 12 Å was used to solve long-range electrostatic interactions. An in-house code which automatically assigns partial charges based on an ab initio Bader charge analysis performed on magnetite surface atoms is used together with a modified CLAYFF parameter set^40,41^ to describe the magnetite-organic interface. It should be noted that for each specific dissociative adsorption motif on magnetite a specific partial charge set is used. Thus, it is implicitly accounted for the rather complex oxidation state of magnetite by considering a different partial charge scheme for magnetite depending on the particular situation.

Parameters and partial point charges used in the empirical force field can be found in Konuk et al.^24^. OLAC and oleate are described by the General AMBER Force Field (GAFF)^42^ and partial charges have been optimized by a two-step restrained electrostatic potential fit charge model (RESP)^43^ to obtain partial point charges for OLAC and oleate which reproduce the electrostatic potential from QM calculations using the B3LYP functional method, a polarizable water model(c-PCM), and a cc-pV(T+d)Z basis set (more details are found in Lundborg and Lindahl^44^). To obtain partial point charges, topologies and corresponding parameters practically, the Small molecule Topology GEnerator (STaGE)^44^ is used, which has a built-in RESP implementation and calculates the desired charges from simple SMILES (Simplified Molecular Input Line Entry Specification). Magnetite surface slabs are generated by replicating a 4 × 4 × 2 bulk unit cell, cleavage and removal of additional iron atoms at vacancies for SCV. Only along *x*- and *y*-directions, periodic boundary conditions are applied.

After the magnetite surfaces have been thermally pretreated at room temperature, OLAC molecules are added iteratively every 100 ps from a random starting position 20 Å away from the magnetite surface with random thermal velocities corresponding to the temperature in the experiments. The carboxylic group of OLAC is pulled to the surface only during the first 7 ps of deposition with a constant force of 0.33 nN using steered MD. The pulling force used in our simulation was carefully chosen and is weaker than in a previous work (0.83 nN in Dietrich et al.^29^) to ensure that the formation of the monolayer is not significantly perturbed. Nevertheless, it could have some influence and explain the higher than experimentally observed coverage of the (001) surface, as the disordered ligand structure is readily penetrated. We assumed in all (001) simulations a distorted, bulk-truncated (DBT) structure as a starting point, although starting from a SCV surface might be a better initial choice in case of no or low ligand surface coverage. We rationalize this decision by previous experimental observations^17,20,21^ stating that the SCV surface is already lifted at very low ligand coverages. After OLAC reaches the magnetite surface it forms hydrogen bonds with suitable surface oxygens. Hydrogen bonds of OLAC to other OLAC or surface oxygens as well as oleates are estimated using a simple distance threshold between acceptor and hydrogen of 2.5 Å, which has already been shown to be sufficient for a similar setup^29,45^. A proton transfer was initiated inspired by the works of refs. Dietrich et al. ^29,45^ and previously adapted for the adsorption of formic acid on magnetite surfaces^24^. Similarly, the proton transfer reaction of the dissociative adsorption is triggered when the distance of a carboxylic hydrogen to a surface oxygen is ≤2.5 Å. Consequently, OLAC is allowed to adsorb and eventually dissociate until a certain coverage is achieved. After reaching a coverage of around 2.23 molecules/nm^2^, the highest observed coverage on both (001) and (111) surfaces in our MD simulations, no further initial (molecular) adsorption of OLAC was observed. Dissociative adsorption of OLAC on Fe_tet1_-terminated magnetite (111) is modeled similarly to the adsorption on a (001) surface. Except that in the case of a (111) surface, we assume that after dissociation of OLAC, the hydrogen continues to migrate to the second nearest oxygen on the magnetite (111) surface after an initial adsorption to the nearest oxygen^23,24^. Analyses for different coverages were carried out on 1 ns long simulations at 300 K picking a snapshot after a certain coverage was reached.

Electron densities are obtained using the Density Profile Tool in VMD^46^ and are averaged over the last 1 ns of the NVT simulations. In order to account for the actual non-point charges in the electron density diagrams, a Gaussian charge smearing is used, where the van der Waals radii of the respective element (i.e. C, O, H, and Fe) are used to smear the atomic point charges, which would otherwise produce a rather artificially noisy signal. Each profile was normalized to one and multiplied with the corresponding theoretical number of total electrons. Individual calculated profiles are combined to obtain the total electron density of the system and are given in *e*/Å^3^ as a function of the distance perpendicular to the surface.

## Supplementary information

Supplementary Information

Description of Additional Supplementary Files

Supplementary Movie 1

Supplementary Movie 2

## Data Availability

A Supplementary Information file is available together with this manuscript. It includes details on the oleic acid dosing setup; full SXRD CTR data sets and tables with fitted atomic positions and occupancies for the clean surfaces and after oleic acid adsorption; XRR fit parameters; STM images after oleic acid adsorption on magnetite (111); additional information on MD simulations and snapshots for different oleic acid coverages; overview of adsorption geometries; formation energies at a given oleate/oleic acid coverage; Supplementary Movies 1 and 2 of the oleic acid layer growth on both surfaces. The data that support the findings of this study are available from the corresponding authors A.S. and R.H.M. upon reasonable request.

## Code availability

Input structures and scripts used for the molecular dynamics simulations are available from the corresponding author R.H.M. upon reasonable request.
