# Supplementary material for: Adsorption of oleic acid on magnetite facets
Source: Commun Chem. 2022 Oct 23;5:134. doi: 10.1038/s42004-022-00741-0 (PMC9814498; doi:10.1038/s42004-022-00741-0)
Supplement: Supplementary file 1 — Supplementary Information [file 42004_2022_741_MOESM1_ESM.pdf]

Supplementary Information: Adsorption of Oleic Acid on Magnetite Facets

Marcus Creutzburg,[†] Mine Konuk,[‡] Steffen Tober,[†,¶] Simon Chung,[†] Björn Arndt,[†] Heshmat Noei,[†] Robert H. Meißner,[*,‡,§] and Andreas Stierle[*,†,¶]

†*Centre for X-Ray and Nano Science CXNS, Deutsches Elektronen-Synchrotron DESY, Notkestr. 85, 22607 Hamburg, Germany*

‡*Institute of Polymers and Composites, Hamburg University of Technology, Denickestr. 15, 21073 Hamburg, Germany*

¶*Department of Physics, University of Hamburg, Luruper Chaussee 149, 22761 Hamburg, Germany*

§*Institute of Surface Science, Helmholtz-Zentrum Hereon, Max-Planck-Str. 1, 21502 Geesthacht, Germany*

E-mail: robert.meissner@tuhh.de; andreas.stierle@desy.de

Supplementary Methods

Oleic acid dosing setup

A customized UHV nozzle was designed to achieve localized dosing of oleic acid under UHV conditions (compare Fig. S1). The dosing nozzle is connected to a gas line which is built from CF16 UHV components and evacuated to 2×10^{-8} mbar. This is in the range of the oleic acid vapor pressure at room temperature[1,2]. Oleic acid was purchased with a purity of $\geq 99.0\%$ The gas line and the oleic acid glass vial are baked at 80 °C by heating tapes attached at the gas line outer walls to remove residual water from the system. The corner valve to the chamber is opened to start dosing. The nozzle itself is moved as close to the sample as possible by a bellow, as shown in Fig. S2. The bellow can then be retracted again for sample transfer. The pressure during dosing is measured with a cold cathode pressure gauge typically 30 cm away from the sample.

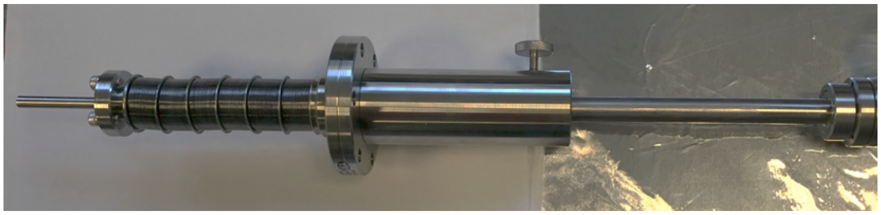

Figure S1: Dosing nozzle for oleic acid with adjustable bellow.

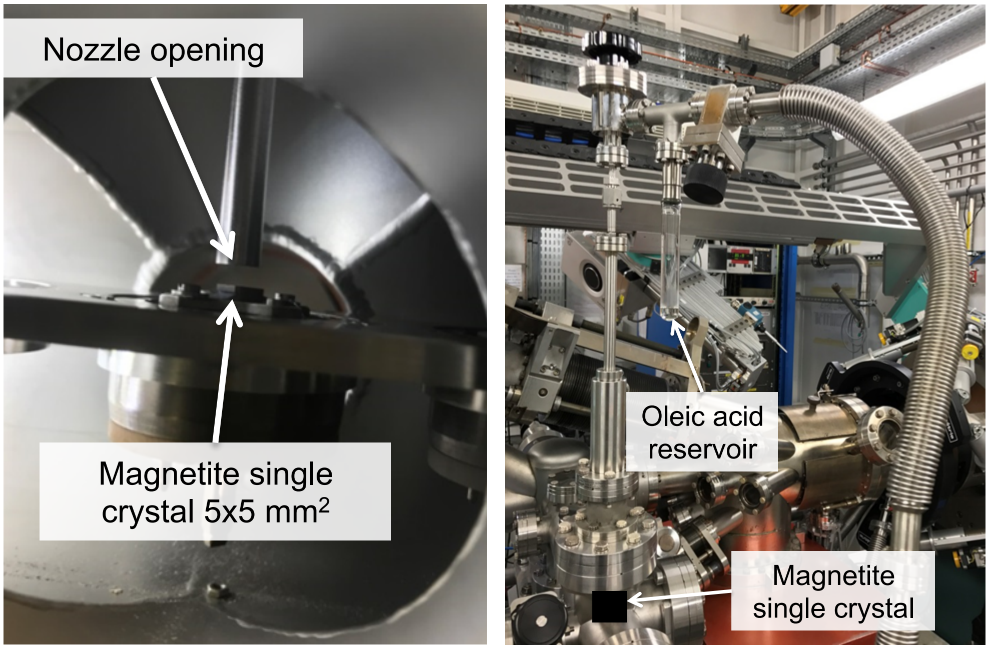

Figure S2: Dosing of oleic acid on a magnetite single crystal at the ID03 beamline, ESRF. Left: The nozzle opening is a few millimeters away from the crystal. Right: The oleic acid gas line was evacuated to 2×10^{-8} mbar using a turbomolecular pump. Oleic acid was dosed from a glass reservoir.

Auger Electron Spectroscopy

The Auger electron spectrum after the adsorption of oleic acid also shows a clear indication for an adsorbed layer on the surface, as exemplary shown for the (111) surface in Fig. S3. The iron and oxygen peaks from the magnetite substrate decrease in intensity and a carbon peak at 272 eV appears[3], which is assigned to aliphatic carbon of oleic acid.

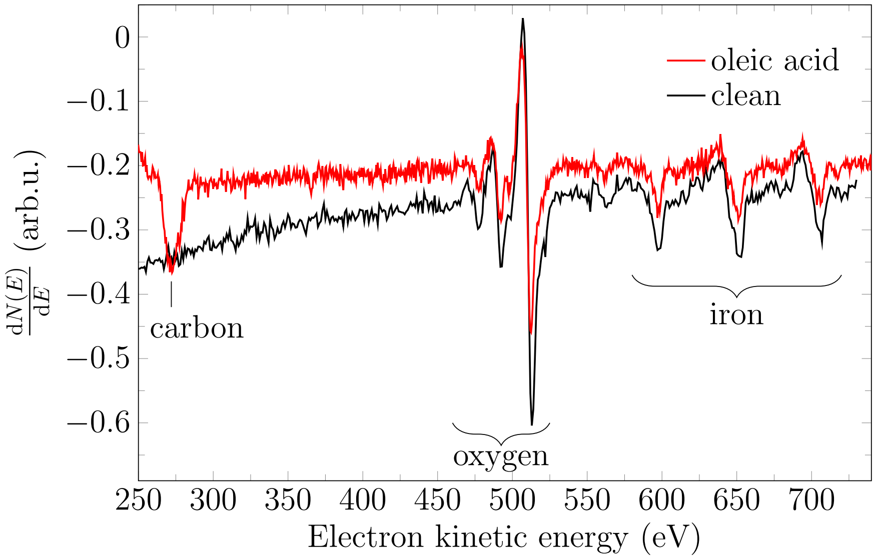

Figure S3: Auger electron spectra of magnetite (111) before and after oleic acid adsorption. The signal $\frac{\mathrm{d}N(E)}{\mathrm{d}E}$ is normalized to the incident electron beam current, which is in the range of $\sim 10-15\,\mu\mathrm{A}$).

Supplementary Notes 1

Surface X-ray Diffraction (SXRD)

The Debye-Waller-factors were fixed to the bulk values $0.49\,\text{Å}^2$ for oxygen, $0.34\,\text{Å}^2$ for tetrahedral and $0.46\,\text{Å}^2$ for octahedral iron[4]. The fits are characterized by the goodness-of-fit, also called reduced χ^2_{red} value, which is defined by:

$$\chi^2_{\mathrm{red}} = \frac{1}{N-P}\sum\frac{(F_{\mathrm{exp}}-F_{\mathrm{theo}})^2}{\sigma^2}, \quad (1)$$

with the experimentally measured structure factor F_{exp}, the theoretical structure factor F_{theo} calculated from the underlying model, the experimental structure factor error σ (estimated to be 15%), the number of data points N and the number of parameters P.

Supplementary Discussion 1

SXRD: Oleic acid adsorption on magnetite (001)

Fig. S4 shows the CTR data obtained for the clean magnetite (001) surface and after oleic acid adsorption.

Fe displacements in the first nine layers were fitted alongside the occupancies of interstitial tetrahedral iron below the surface, the octahedral vacancy sites and adjacent octahedral iron in the same layer and the carboxylic group. An occupancy parameter was assigned for each atomic layer that accounts for the average probability to find the respective sites in this layer occupied. The error bars on the occupancies are 0.20 for oxygen and 0.05 for iron[5–7]. The occupancy bars in Fig. 1c of the main article that are not labeled with an occupancy value were fixed during the refinement. For the fit of the oleic acid covered surface also in-plane and z-displacements of the oxygen atoms were allowed, see Table S3.

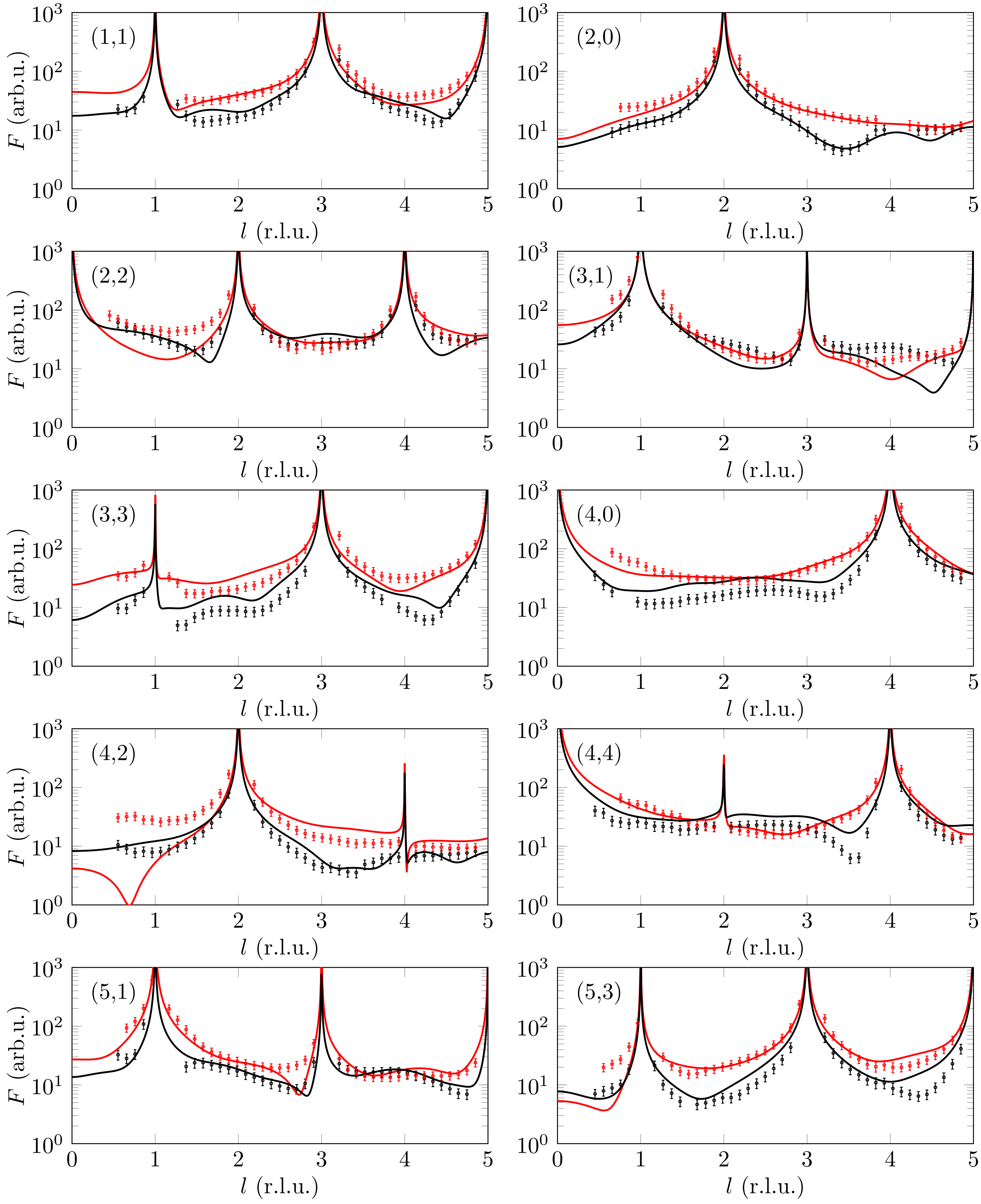

Figure S4: Structure factors of clean magnetite (001) surface (data points and fit: black, $\chi^2_{\text{red}} = 1.71$) and after oleic acid adsorption (data points and fit in red, $\chi^2_{\text{red}} = 1.26$)

Table S1: z-displacements and occupancies of the first two iron layers on the clean magnetite (001) and after oleic acid adsorption at room temperature obtained from the SXRD fits. Also shown are fitting parameters obtained for the formic acid covered surface[8]. All displacement values are given as the difference from the bulk atom position. For error bars see Tables S2 and S3.

	**clean surface**		**oleic acid RT**		**50 L formic acid RT[8]**	
atomic layer	z disp./Å	occ.	z disp./Å	occ.	z disp./Å	occ.
OCO				1.0		1.7
top layer Fe_{oct}	−0.296	1.00	−0.011	1(fixed)	+0.063	1(fixed)
additional Fe_{tet}	+0.047	1.00	−0.061	0.11	−0.001	0.09
Fe_{oct} vacancy sites		0.01	−0.041	1.00	−0.026	0.95
Fe_{oct} adjacent to vacancies	−0.069	1.00	−0.041	0.96	−0.026	0.90

Table S2: Atom positions and occupancies of clean magnetite (001) obtained from the SXRD fit. Atoms are sorted with decreasing z coordinates. All coordinates are given relative to the unit cell dimensions $a_0 = 8.394$ Å. Error bars are given in absolute values (Å). Occupancies given as '1' are not fitted.

atom	x	y	z	occ.
O	0.9830	0.4905	2.7442	1
O	0.2595	0.7670	2.7442	1
O	0.4905	0.9830	2.7442	1
O	0.7670	0.2595	2.7442	1
Fe	0.4910±0.0827	0.7463±0.0839	2.7147±0.0214	1
Fe	0.2590±0.0827	0.0037±0.0839	2.7147±0.0214	1
Fe	0.0037±0.0839	0.2590±0.0827	2.7147±0.0214	1
Fe	0.7463±0.0839	0.4910±0.0827	2.7147±0.0214	1
O	0.2422	0.2422	2.7454	1
O	0.0285	0.0285	2.7239	1
O	0.5078	0.5078	2.7454	1
O	0.7215	0.7215	2.7239	1
Fe	0.6363±0.0250	0.1137±0.0250	2.6353±0.0167	1
Fe	0.1137±0.0250	0.6363±0.0250	2.6353±0.0167	1
Fe	0.3750	0.3750	2.6306±0.0459	1.00 ± 0.05
O	0.5082	0.7586	2.4873	1
O	-0.0086	0.2418	2.4873	1
O	0.7586	0.5082	2.4873	1
O	0.2418	-0.0086	2.4873	1
Fe	0.7433±0.1519	0.7433±0.1519	2.4918±0.0872	1.00 ± 0.05
Fe	0.0067±0.1519	0.0067±0.1519	2.4918±0.0872	1.00 ± 0.05
Fe	0.2503±0.1519	0.2503±0.1519	2.4950±0.0390	0.01 ± 0.05
Fe	0.4997±0.1519	0.4997±0.1519	2.4950±0.0390	0.01 ± 0.05
O	0.7624	0.9876	2.4885	1
O	0.2405	0.5095	2.4986	1
O	0.9876	0.7624	2.4885	1
O	0.5095	0.2405	2.4986	1
Fe	0.8745±0.0235	0.3739±0.0231	2.3659±0.0230	1
Fe	0.3739±0.0231	0.8745±0.0235	2.3659±0.0230	1
O	0.4909	0.4909	2.2411	1
O	0.7527	0.7527	2.2494	1
O	0.9973	0.9973	2.2494	1
O	0.2591	0.2591	2.2411	1
Fe	0.4931±0.0157	0.2569±0.0157	2.2723±0.0138	1
Fe	0.7528±0.0172	-0.0028±0.0172	2.2479±0.0138	1
Fe	0.2569±0.0157	0.4931±0.0157	2.2723±0.0138	1
Fe	-0.0028±0.0172	0.7528±0.0172	2.2479±0.0138	1
O	0.0096	0.5044	2.2357	1
O	0.5044	0.0096	2.2357	1
O	0.7404	0.2456	2.2357	1
O	0.2456	0.7404	2.2357	1
Fe	0.1276±0.0282	0.1276±0.0282	2.1241±0.0158	1
Fe	0.6224±0.0282	0.6224±0.0282	2.1241±0.0158	1
O	0.0054	0.7447	2.0033	1
O	0.7447	0.0054	2.0033	1
O	0.2456	0.5044	2.0021	1
O	0.5044	0.2456	2.0021	1
Fe	0.0021±0.0205	0.4981±0.0196	1.9972±0.0126	1
Fe	0.7479±0.0205	0.2519±0.0196	1.9972±0.0126	1
Fe	0.2519±0.0196	0.7479±0.0205	1.9972±0.0126	1
Fe	0.4981±0.0196	0.0021±0.0205	1.9972±0.0126	1
O	0.9923	0.2528	1.9961	1
O	0.2528	0.9923	1.9961	1
O	0.4972	0.7577	1.9961	1
O	0.7577	0.4972	1.9961	1
Fe	0.3750	0.3750	1.8779±0.0152	1
Fe	0.8750	0.8750	1.8731±0.0152	1
O	0.9949	0.4950	1.7529	1
O	0.2550	0.7551	1.7529	1
O	0.4950	0.9949	1.7529	1
O	0.7551	0.2550	1.7529	1
Fe	0.5000	0.7501	1.7468±0.0105	1
Fe	0.2500	0.0000	1.7468±0.0105	1
Fe	0.0000	0.2500	1.7468±0.0105	1
Fe	0.7501	0.5000	1.7468±0.0105	1
O	0.2449	0.2449	1.7476	1
O	0.0051	0.0051	1.7440	1
O	0.5051	0.5051	1.7476	1
O	0.7449	0.7449	1.7440	1
Fe	0.6250	0.1250	1.6287	1
Fe	0.1250	0.6250	1.6287	1
O	0.5051	0.7450	1.5008	1
O	0.0050	0.2449	1.5008	1
O	0.7450	0.5051	1.5008	1
O	0.2449	0.0050	1.5008	1
Fe	0.7500	0.7500	1.4994	1
Fe	0.0000	0.0000	1.4994	1
Fe	0.5000	0.5000	1.5000	1
Fe	0.2500	0.2500	1.5000	1
O	0.7550	0.9950	1.4883	1
O	0.2550	0.4950	1.4924	1
O	0.9950	0.7550	1.4883	1
O	0.4950	0.2550	1.4924	1
Fe	0.8749	0.3750	1.3725	1
Fe	0.3750	0.8749	1.3725	1
O	0.4879	0.4879	1.2540	1
O	0.7621	0.7621	1.2540	1
O	0.9879	0.9879	1.2540	1
O	0.2621	0.2621	1.2540	1
Fe	0.5000	0.2500	1.2500	1
Fe	0.7500	0.0000	1.2500	1
Fe	0.2500	0.5000	1.2500	1
Fe	0.0000	0.7500	1.2500	1

Table S3: Atom positions and occupancies of oleic acid on magnetite (001) obtained from the SXRD fit. Atoms are sorted with decreasing z coordinates. All coordinates are given relative to the unit cell dimensions $a_0 = 8.394\,\text{Å}$. Error bars are given in absolute values (Å). Occupancies given as '1' are not fitted.

atom	x	y	z	occupancy
C	0.3750±0.0670	0.8750±0.0670	2.0670±0.1628	1.00 ± 0.20
O	0.4878±0.0670	0.7622±0.0670	1.9501±0.0998	1.00 ± 0.20
O	0.2622±0.0670	-0.0122±0.0670	1.9501±0.0998	1.00 ± 0.20
O	0.0059±0.0224	0.5059±0.0224	1.7713±0.0658	1
O	0.2441±0.0224	0.7441±0.0224	1.7713±0.0658	1
O	0.5059±0.0224	0.0059±0.0224	1.7713±0.0658	1
O	0.7441±0.0224	0.2441±0.0224	1.7713±0.0658	1
Fe	0.4919±0.0055	0.7581±0.0055	1.7487±0.0123	1
Fe	0.2581±0.0055	-0.0081±0.0055	1.7487±0.0123	1
Fe	-0.0081±0.0055	0.2581±0.0055	1.7487±0.0123	1
Fe	0.7581±0.0055	0.4919±0.0055	1.7487±0.0123	1
O	0.2385±0.0236	0.2385±0.0236	1.6993±0.0378	1
O	0.0115±0.0236	0.0115±0.0236	1.6993±0.0378	1
O	0.5115±0.0236	0.5115±0.0236	1.6993±0.0378	1
O	0.7385±0.0236	0.7385±0.0236	1.6993±0.0378	1
Fe	0.6250	0.1250	1.6177±0.0186	1
Fe	0.1250	0.6250	1.6177±0.0186	1
Fe	0.3750	0.3750	1.6177±0.0186	0.11 ± 0.05
O	0.5032±0.0732	0.7468±0.0732	1.4991±0.0384	1
O	0.0210±0.0732	0.2290±0.0732	1.4991±0.0384	1
O	0.7290±0.0732	0.5210±0.0732	1.4991±0.0384	1
O	0.2468±0.0732	0.0032±0.0732	1.4991±0.0384	1
Fe	0.7500±0.0062	0.7500±0.0062	1.4951±0.0110	0.96 ± 0.05
Fe	0.2500±0.0062	0.2500±0.0062	1.4951±0.0110	1.00 ± 0.05
Fe	0.5000±0.0062	0.5000±0.0062	1.4951±0.0110	1.00 ± 0.05
Fe	0.0000±0.0062	0.0000±0.0062	1.4951±0.0110	0.96 ± 0.05
O	0.7552±0.0302	0.9948±0.0302	1.4981±0.0352	1
O	0.2552±0.0302	0.4948±0.0302	1.4981±0.0352	1
O	0.9948±0.0302	0.7552±0.0302	1.4981±0.0352	1
O	0.4948±0.0302	0.2552±0.0302	1.4981±0.0352	1
Fe	0.8750	0.3750	1.3716±0.0137	1
Fe	0.3750	0.8750	1.3716±0.0137	1
O	0.4968±0.0219	0.4968±0.0219	1.2580±0.0408	1
O	0.7532±0.0219	0.7532±0.0219	1.2580±0.0408	1
O	0.9968±0.0219	0.9968±0.0219	1.2580±0.0408	1
O	0.2532±0.0219	0.2532±0.0219	1.2580±0.0408	1
Fe	0.4986±0.0071	0.2514±0.0071	1.2464±0.0102	1
Fe	0.7514±0.0071	-0.0014±0.0071	1.2464±0.0102	1
Fe	0.2514±0.0071	0.4986±0.0071	1.2464±0.0102	1
Fe	-0.0014±0.0071	0.7514±0.0071	1.2464±0.0102	1
O	0.0050±0.0196	0.5050±0.0196	1.2293±0.0396	1
O	0.5050±0.0196	0.0050±0.0196	1.2293±0.0396	1
O	0.7450±0.0196	0.2450±0.0196	1.2293±0.0396	1
O	0.2450±0.0196	0.7450±0.0196	1.2293±0.0396	1
Fe	0.1250	0.1250	1.1225±0.0109	1
Fe	0.6250	0.6250	1.1225±0.0109	1
O	0.0121	0.7379	1.0040	1
O	0.7379	0.0121	1.0040	1
O	0.2379	0.5121	1.0040	1
O	0.5121	0.2379	1.0040	1
Fe	-0.0004±0.0047	0.4996±0.0047	0.9975±0.0081	1
Fe	0.7504±0.0047	0.2504±0.0047	0.9975±0.0081	1
Fe	0.2504±0.0047	0.7504±0.0047	0.9975±0.0081	1
Fe	0.4996±0.0047	-0.0004±0.0047	0.9975±0.0081	1
O	0.9879	0.2621	0.9960	1
O	0.2621	0.9879	0.9960	1
O	0.4879	0.7621	0.9960	1
O	0.7621	0.4879	0.9960	1
Fe	0.3750	0.3750	0.8734±0.0082	1
Fe	0.8750	0.8750	0.8734±0.0082	1

Supplementary Notes 2

XRR

Table S4: Fit parameters of XRR data analysis of clean magnetite (001) and oleic acid on magnetite (001) obtained from Fewlay.

Parameter	Clean surface	Oleic acid
d_{OA} (Å)		8.18
σ_{OA} (Å)		2.45
$2\delta_{\mathrm{OA}}$		1.03E−06
$2\beta_{\mathrm{OA}}$		1.16E−09
$d_{\text{mid-layer}}$ (Å)	2.82	2.76
$\sigma_{\text{mid-layer}}$ (Å)	1.62	2.01
$2\delta_{\text{mid-layer}}$	4.85E−06	4.88E−06
$2\beta_{\text{mid-layer}}$	3.662E−07	2.70E−07
$\sigma_{\text{substrate}}$ (Å)	10.5	11.9
χ^2_{norm}	0.05	0.05
ρ_{OA} (e/Å^3)		0.15
Molecules/unit cell		0.54
Molecules/nm^2		0.76
$\rho_{\text{mid-layer}}$ (e/Å^3)	0.69	0.69
$2\delta^{\mathrm{OA}}_{\mathrm{RT}} = 2.61\mathrm{E}{-6}\ (0.37\,e/\text{Å}^3)$		
$2\delta^{\mathrm{magn}}_{\mathrm{bulk}} = 1.0575\mathrm{E}{-5}\ (1.503\,e/\text{Å}^3)$		
$2\beta^{\mathrm{magn}}_{\mathrm{bulk}} = 3.662\mathrm{E}{-7}$		

Supplementary Discussion 2

SXRD: Oleic acid adsorption on magnetite (111)

Oleic acid on the surface was modeled as on the magnetite (111) surface with an OCO carboxylic end group. It was placed in the quasi-bidentate adsorption site before starting the fit. The refinement includes occupancies and z-displacements for the carboxylic group and the first seven atomic layers. The refined model obtained from formic acid on magnetite (111) was chosen as a starting model for the fit[9]. In addition, the carboxylic group was allowed to be displaced in the in-plane direction (in x and y) to cover the potential chelating adsorption site. However, the influence on the in-plane displacement of the end group is not significant and the OCO group stayed in the quasi-bidentate adsorption site during the fit. The experimental CTR data for the clean (111) surface and after oleic acid adsorption along with the best fit are shown in Fig. S5.

The occupancy profile in Fig. 1f in the main article shows an OCO coverage of 18% which is less than the coverage determined by XRR but can be assigned to the low scattering contribution of carbon and oxygen (typical error bars $\pm$20%) and the disorder of the molecules. The first six layers below the surface show a reduced occupancy for both iron (error bars $\pm$5%) and oxygen, which suggests a roughness increase and can be explained by the increased amount of hydrogen present from the dissociation of oleic acid. The surface roughening on the atomic scale is expected to be similar to the formic acid-induced roughening on magnetite (111)[9]. Due to the increased amount of atomic hydrogen which adsorbs at surface oxygen, recombined OH or water can desorb from the surface while iron ions diffuse into the bulk. The adsorption of hydrogen has already been proven as a reason for an atomic roughening on the magnetite (001) surface[10]. The following Table S5 summarizes the occupancy and z-displacement parameters for the clean (111) surface and after oleic acid adsorption.

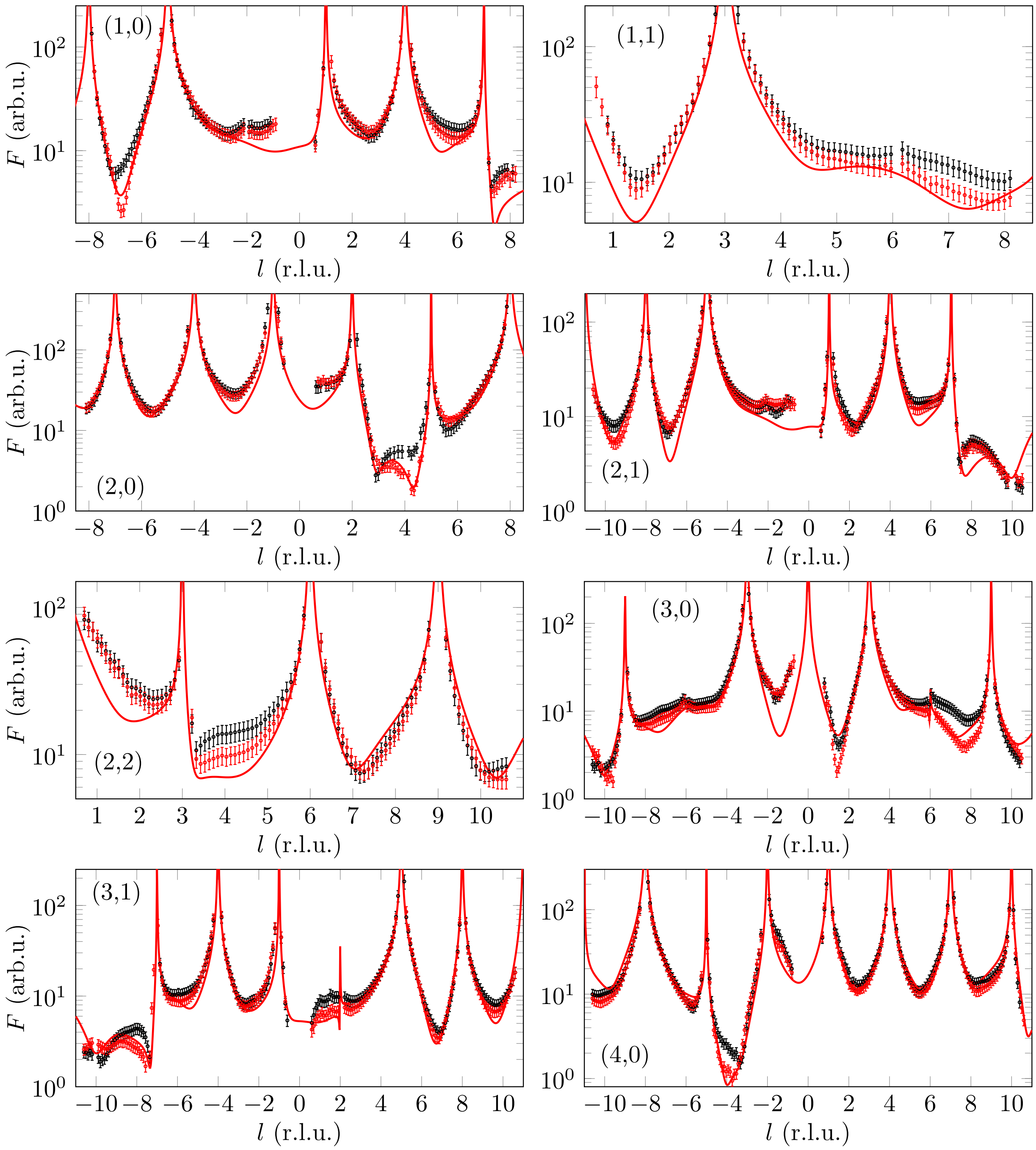

Figure S5: Structure factor data of oleic acid on magnetite (111) (red dots). Also shown are the best fit $\chi_{\text{red}} = 3.67$ (red line) and the clean surface data (black).

Table S5: Fit parameters of the clean (111) surface (UHV annealing), after oleic acid adsorption at room temperature. All displacement values are given as the difference from the bulk atom positions. For error bars see Tables S6 and S7.

	**clean surface**		**oleic acid RT**	
atomic layer	z disp./Å	occ.	z disp./Å	occ.
COO				0.18
tet1 (surface)	−0.134	0.80	−0.079	0.72
O1	−0.186	1 (fixed)	−0.214	0.72
oct1	+0.054	0.89	+0.033	0.80
O2	−0.061	1 (fixed)	−0.084	0.82
tet2	−0.020	0.94	−0.013	0.86
oct2	−0.017	0.96	−0.023	0.86
tet1 (bulk)	−0.020	1.00	−0.047	0.92

Table S6: Atom positions and occupancies of the clean magnetite (111) surface obtained from the SXRD fit. All coordinates are given relative to the unit cell dimensions $a_1 = a_2 = 5.935$ Å and $a_3 = 14.539$ Å. Error bars are given in absolute values (Å). Atoms are sorted with increasing z coordinates. Relaxations in z were allowed for atoms with $z \geq 1.6236$. Atom occupancies given as '1' were not fitted.

atom	x	y	z	occupancy
Fe	0.0000	0.0000	1.0000	1
Fe	0.3333	0.6667	1.0417	1
O	0.1601	0.8399	1.0850	1
O	0.1601	0.3203	1.0850	1
O	0.6797	0.8399	1.0850	1
O	0.6667	0.3333	1.0882	1
Fe	0.3333	0.1667	1.1667	1
Fe	0.8333	0.6667	1.1667	1
Fe	0.8333	0.1667	1.1667	1
O	0.0000	1.0000	1.2451	1
O	-0.0131	0.4935	1.2484	1
O	0.5065	1.0131	1.2484	1
O	0.5065	0.4935	1.2484	1
Fe	0.3333	0.6667	1.2917	1
Fe	0.6667	0.3333	1.3333	1
Fe	1.0000	1.0000	1.3750	1
O	0.3333	0.6667	1.4216	1
O	0.3464	0.1732	1.4183	1
O	0.8268	0.6536	1.4183	1
O	0.8268	0.1732	1.4183	1
Fe	0.0000	0.5000	1.5000	1
Fe	0.5000	1.0000	1.5000	1

atom	x	y	z	occupancy
Fe	0.5000	0.5000	1.5000	1
Fe	0.0000	1.0000	1.5000	1
O	0.1732	0.8268	1.5817	1
O	0.1732	0.3464	1.5817	1
O	0.6536	0.8268	1.5817	1
O	0.6667	0.3333	1.5784	1
Fe	0.0000	1.0000	1.6236 ± 0.0021	1.00 ± 0.05
Fe	0.3333	0.6667	1.6655 ± 0.0026	0.96 ± 0.05
Fe	0.6667	0.3333	1.7069 ± 0.0025	0.94 ± 0.05
O	0.0000	1.0000	1.7507 ± 0.0078	1
O	0.0131	0.5065	1.7474 ± 0.0078	1
O	0.4935	0.9869	1.7474 ± 0.0078	1
O	0.4935	0.5065	1.7474 ± 0.0078	1
Fe	0.1667	0.8333	1.8370 ± 0.0021	0.89 ± 0.05
Fe	0.1667	0.3333	1.8370 ± 0.0021	0.89 ± 0.05
Fe	0.6667	0.8333	1.8370 ± 0.0021	0.89 ± 0.05
O	0.3333	0.6667	1.8990 ± 0.0045	1
O	0.3203	0.1601	1.9022 ± 0.0045	1
O	0.8399	0.6797	1.9022 ± 0.0045	1
O	0.8399	0.1601	1.9022 ± 0.0045	1
Fe	0.6667	0.3333	1.9491 ± 0.0035	0.80 ± 0.05

Table S7: Atom positions and occupancies of oleic acid on magnetite (111) obtained from SXRD fit. All coordinates are given relative to the unit cell dimensions $a_1 = a_2 = 5.935$ Å and $a_3 = 14.539$ Å. Error bars are given in absolute values (Å). Atoms are sorted with increasing z coordinates. Relaxations in z were allowed for atoms with $z \geq 1.6218$. Atom occupancies given as '1' were not fitted.

atom	x	y	z	occupancy
Fe	0.0000	0.0000	1.0000	1
Fe	0.3333	0.6667	1.0417	1
O	0.1601	0.8399	1.0850	1
O	0.1601	0.3203	1.0850	1
O	0.6797	0.8399	1.0850	1
O	0.6667	0.3333	1.0882	1
Fe	0.3333	0.1667	1.1667	1
Fe	0.8333	0.6667	1.1667	1
Fe	0.8333	0.1667	1.1667	1
O	0.0000	1.0000	1.2451	1
O	-0.0131	0.4935	1.2484	1
O	0.5065	1.0131	1.2484	1
O	0.5065	0.4935	1.2484	1
Fe	0.3333	0.6667	1.2917	1
Fe	0.6667	0.3333	1.3333	1
Fe	1.0000	1.0000	1.3750	1
O	0.3333	0.6667	1.4216	1
O	0.3464	0.1732	1.4183	1
O	0.8268	0.6536	1.4183	1
O	0.8268	0.1732	1.4183	1
Fe	0.0000	0.5000	1.5000	1
Fe	0.5000	1.0000	1.5000	1
Fe	0.5000	0.5000	1.5000	1
Fe	0.0000	1.0000	1.5000	1
atom	x	y	z	occupancy
O	0.1732	0.8268	1.5817	1
O	0.1732	0.3464	1.5817	1
O	0.1732	0.3464	1.5817	1
O	0.6536	0.8268	1.5817	1
O	0.6667	0.3333	1.5784	1
Fe	0.0000	1.0000	1.6218 ± 0.0020	0.92 ± 0.05
Fe	0.3333	0.6667	1.6651 ± 0.0024	0.86 ± 0.05
Fe	0.6667	0.3333	1.7074 ± 0.0024	0.86 ± 0.05
O	0.0000	1.0000	1.7491 ± 0.0073	0.82 ± 0.20
O	0.0131	0.5065	1.7458 ± 0.0073	0.82 ± 0.20
O	0.4935	0.9869	1.7458 ± 0.0073	0.82 ± 0.20
O	0.4935	0.5065	1.7458 ± 0.0073	0.82 ± 0.20
Fe	0.1667	0.8333	1.8356 ± 0.0017	0.80 ± 0.05
Fe	0.1667	0.3333	1.8356 ± 0.0017	0.80 ± 0.05
Fe	0.6667	0.8333	1.8356 ± 0.0017	0.80 ± 0.05
O	0.3333	0.6667	1.9017 ± 0.0046	0.72 ± 0.20
O	0.3203	0.1601	1.9003 ± 0.0046	0.72 ± 0.20
O	0.8399	0.6797	1.9003 ± 0.0046	0.72 ± 0.20
O	0.8399	0.1601	1.9003 ± 0.0046	0.72 ± 0.20
Fe	0.6667	0.3333	1.9529 ± 0.0033	0.72 ± 0.05
O	0.3702 ± 0.0345	0.5901 ± 0.0495	2.0226 ± 0.0351	0.18 ± 0.20
O	0.6307 ± 0.0345	0.3715 ± 0.0495	2.0226 ± 0.0351	0.18 ± 0.20
C	0.5005 ± 0.0345	0.4808 ± 0.0495	2.0515 ± 0.0351	0.18 ± 0.20

Supplementary Notes 3

XRR

Table S8: Fit parameters of XRR data analysis of clean magnetite (111) and oleic acid on magnetite (111) obtained from Fewlay.

Parameter	Clean surface	Oleic acid
d_{OA} (Å)		14.065
σ_{OA} (Å)		2.0224
$2\delta_{\mathrm{OA}}$		1.8321E−06
$2\beta_{\mathrm{OA}}$		1.1357E−09
$d_{\text{mid-layer}}$ (Å)	5.8803	6.5493
$\sigma_{\text{mid-layer}}$ (Å)	2.1835	2.9933
$2\delta_{\text{mid-layer}}$	8.0666E−06	8.6846E−06
$2\beta_{\text{mid-layer}}$	3.6622E−07	2.7076E−07
$\sigma_{\mathrm{substrate}}$ (Å)	3.0381	2.3549
χ^2_{norm}	0.06	0.05
ρ_{OA} (e/Å^3)		0.260
Molecules/unit cell		0.71
Molecules/nm^2		2.33
$\rho_{\text{mid-layer}}$ (e/Å^3)	1.147	1.235
$2\delta^{\mathrm{OA}}_{\mathrm{RT}} = 2.61\mathrm{E}{-6}$ (0.37 e/Å^3)		
$2\delta^{\mathrm{magn}}_{\mathrm{bulk}} = 1.0575\mathrm{E}{-5}$ (1.5032 e/Å^3)		
$2\beta^{\mathrm{magn}}_{\mathrm{bulk}} = 3.662\mathrm{E}{-7}$		

Supplementary Notes 4

Surface morphology after oleic acid adsorption on magnetite (111) determined by STM

The oleic acid covered magnetite (111) surface was investigated with STM at room temperature using a tungsten tip in constant current mode. The base pressure during the measurement was $5 \cdot 10^{-11}$ mbar. The oleic acid was dosed at room temperature and not annealed. The images in Figs. S6 and S7 show the surface after an exposure of oleic acid at $8 \cdot 10^{-7}$ mbar for one hour. Larger terraces are visible which have an average step height of 4.7 ± 0.2 Å. This is the same as for the clean surface (see SI of ref. 11) and proves that there is no termination change upon oleic acid adsorption. Due to the interaction of the tip with the weakly bound, long carbon chain of the molecule the step edges are smeared out.

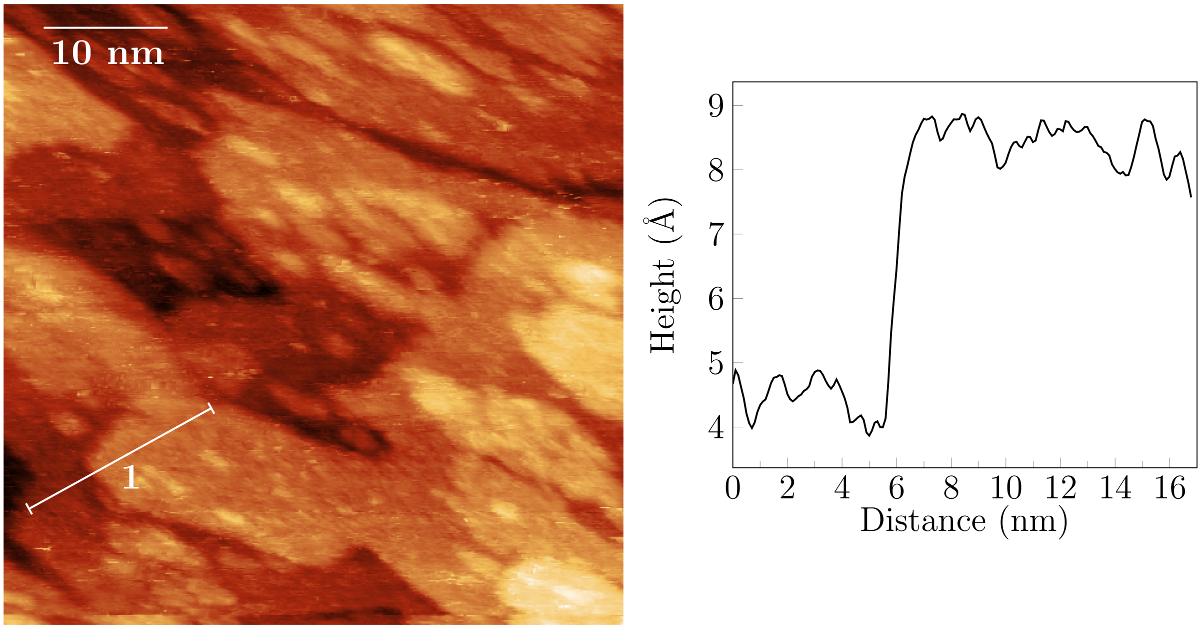

Figure S6: $50 \times 50\,nm^2$ STM image the oleic acid on magnetite (111). Tunneling parameters: 0.5 nA, 1 V. A line scan across a step edge is indicated by the profile on the right.

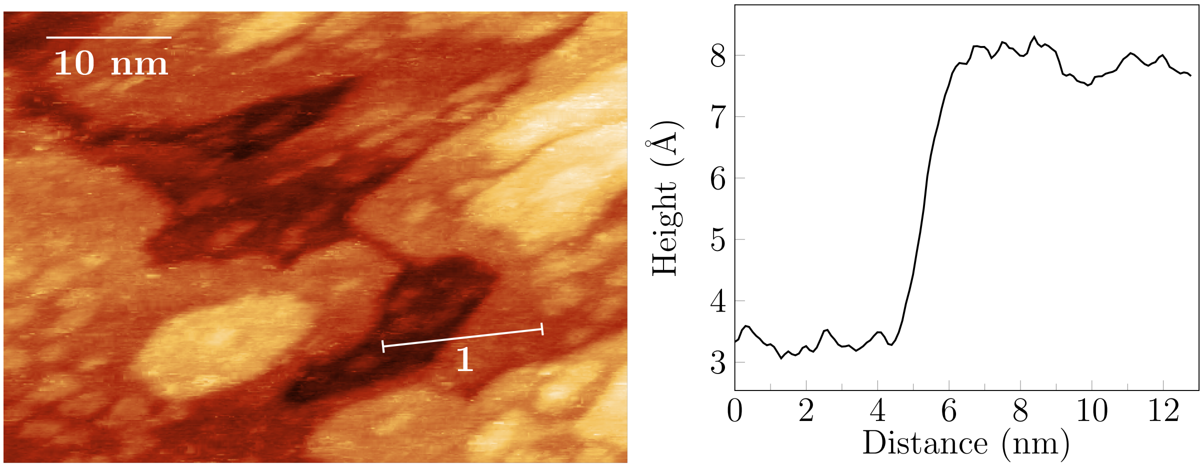

Figure S7: $50 \times 35\,\mathrm{nm}^2$ STM image of oleic acid on magnetite (111). Tunneling parameters: 0.5 nA, 1 V. A line scan across a step edge is indicated by the profile on the right.

Supplementary Discussion 3

Molecular dynamics simulations: Adsorption motifs of oleate/oleic acid on magnetite

To understand the resulting geometries of adsorbed oleic acid, the adsorption modes on magnetite surfaces were studied using MD simulations. In case of molecular adsorption (structure I in Fig. S8a), hydrogen (H_A) of the oleic acid carboxylic end group interacts with the surface in a non-covalent adsorption through a hydrogen bond to the surface oxygen O_M with 2.5 Å O_M–H_A distance. This distance was chosen to trigger the dissociation reaction of oleic acid to form oleate on (001) and (111) magnetite surface in the MD simulations. The H^+ proton is transferred to a nearby O_M and oleate forms automatically a bridging bidentate with two Fe_{oct} on the (001) surface (structure II in Fig. S8a). With increasing coverage, mixed adsorption was observed with a combination of molecular and dissociative adsorption on magnetite (001) and (111). In the case of mixed adsorption, as shown in the upper and lower right panels of Fig. S8, the orientation of the carboxyl groups with respect to the two magnetite surfaces studied, *i.e.*, (001) and (111), differs from those observed in the case of purely molecular or dissociative adsorption. First of all, the hydrogen of the non-dissociated carboxyl groups of oleic acid on both surfaces is directed to another oxygen of an oleate and not to a surface oxygen or hydroxyl. However, the adsorption structure of the non-dissociated carboxyl groups is different on the (111) surface, where the C=O bond lies almost flat on the surface, whereas on the (001) surface the C=O group is more perpendicular to the surface.

To keep track of the energetics involved in forming a monolayer of oleate/oleic acid on magnetite, dissociative adsorption of formic acid serves as a prototypical proton transfer reaction to correct the force field energy for this chemical reaction; similar to Dietrich et al.[12], where methylphosphonic acid on alumina was used instead of octadecylphosphonic acid. It is known from Arndt et al.[8] that formic acid binds strongly to magnetite (001) in a bidentate

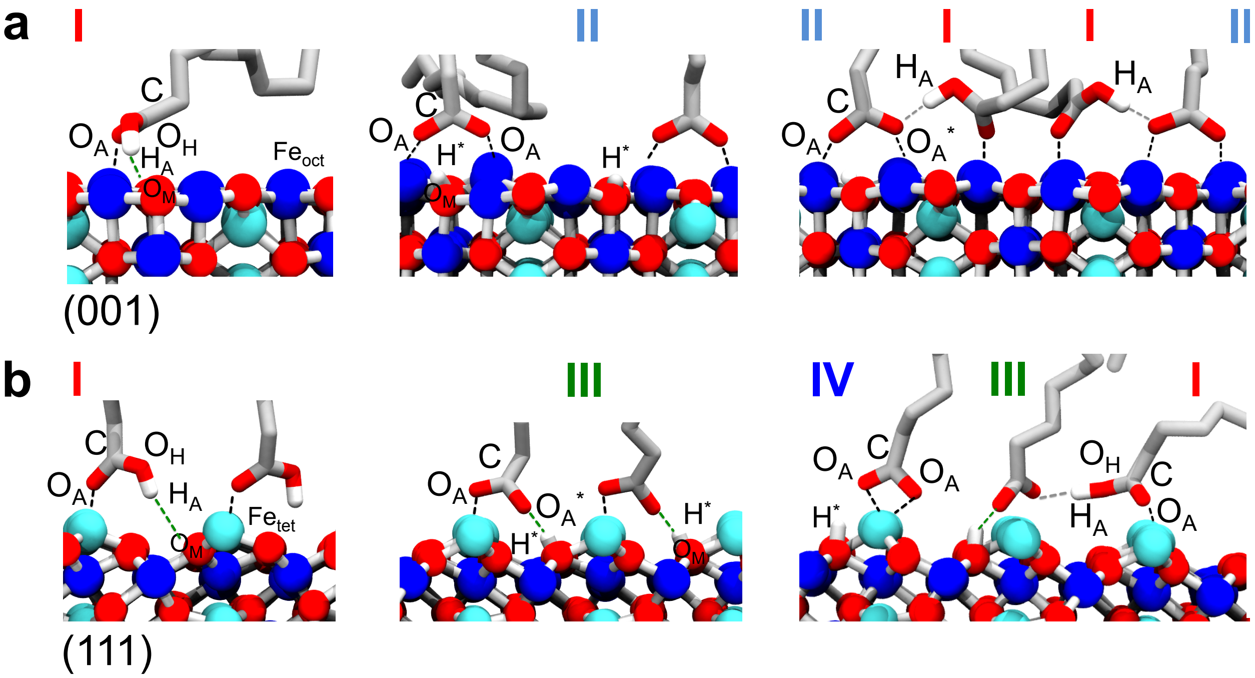

Figure S8: Adsorption structures of oleate/oleic acid on (a) magnetite (001) and (b) magnetite (111). Structure I in both cases is an initially molecularly adsorbed oleic acid (*i.e.* prior to dissociation) observed on both a (001) surface with bulk termination and a (111) surface with Fe_{tet1} termination. Structure II is a dissociatively adsorbed oleate in a bridging bidentate configuration where the two oxygen atoms of the oleate (O_A) are connected to two Fe_{oct} atoms on the (001) surface. Structure III is a quasi-bidentate binding in which one of the oxygen atoms (O_A) is connected to a Fe_{tet1} and the other (O_A^*) is connected to the second nearest surface hydroxyl group (the H^*O_M group) via a hydrogen bond. Structure IV is a chelate where both oxygen atoms of the oleic acid (O_A) are bound to one Fe_{tet} atom. Shown on the right in (a) and (b) is a mixed adsorption mode on a bulk terminated (001) and a Fe_{tet1}-terminated (111).

fashion. The situation is somewhat trickier on (111), where formate can adopt different adsorption motifs depending on the coverage[11]. Therefore, the adsorption energies derived from DFT depend on the particular surface and adsorption motif (*i.e.*, quasi-bidentate, bidentate, chelating or molecular), which makes the corresponding *exact* quantum chemical (QM) correction to our force field energies currently quite inaccessible. Nevertheless, we tried to keep track of the formation energy by using the QM energy change involved in the dissociative adsorption of formic acid on magnetite at 1/2 coverage on (111), $E_{\text{ad}}^{\text{QM}} = -1.83\,\text{eV/formate}$, and 1/3 coverage on (001), $E_{\text{ad}}^{\text{QM}} = -2.30\,\text{eV/formate}$, from QM reference calculations available from previous works[8,9]. Using the force field energy of a single formic acid molecule in vacuum as a reference, $E_{\text{formic}}^{\text{MM}} = 0.075\,\text{eV}$ (no periodic box and consequently no Ewald summation was used in this case), the quantum chemical energy correction of the force field for one proton transfer reaction, following the recipe in ref. 12, is approximately:

$$E_{\text{corr}} \approx E_{\text{ad}}^{\text{QM}}(\lambda) - \left[E^{\text{MM}}(\lambda) - \left(E^{\text{MM}}(\lambda = 0) + E_{\text{formic}}^{\text{MM}}\right)\right] \tag{2}$$

where λ denotes the surface coverage, which is either 1/2 in case of a (111) or 1/3 in case of a (001) magnetite surface. $E^{\text{MM}}(\lambda)$ is the potential energy per formate of the covered surface from the force field and $E^{\text{MM}}(\lambda = 0)$ denotes an empty magnetite slab. Using this correction, the dissociative adsorption energy of one oleic acid dissociatively adsorbing on a magnetite (001) surface is $-2.98\,\text{eV}$ and on a magnetite (111) surface it is $-2.31\,\text{eV}$. The aliphatic tail of the oleic acid molecule thus contributes about $0.5 - 0.7\,\text{eV}$ to the adsorption energy, which is of the same order of magnitude as the value of $1.2\,\text{eV}$ found by Dietrich et al.[12] for octadecylphosphone on alumina. Consequently, the corrected force field adsorption energies given in Fig. S9 for different oleate/oleic acid coverages are estimated from

$$\tilde{E}_{\text{ad}}(\lambda) = E^{\text{MM}}(\lambda) - \left[(E^{\text{MM}}(\lambda = 0) + N(\lambda) \cdot E_{\text{oleic}}^{\text{MM}}\right] + N(\lambda) \cdot E_{\text{corr}} \tag{3}$$

Note that here $E^{\text{MM}}(\lambda)$ is the total potential energy of the oleate/oleic acid covered mag-

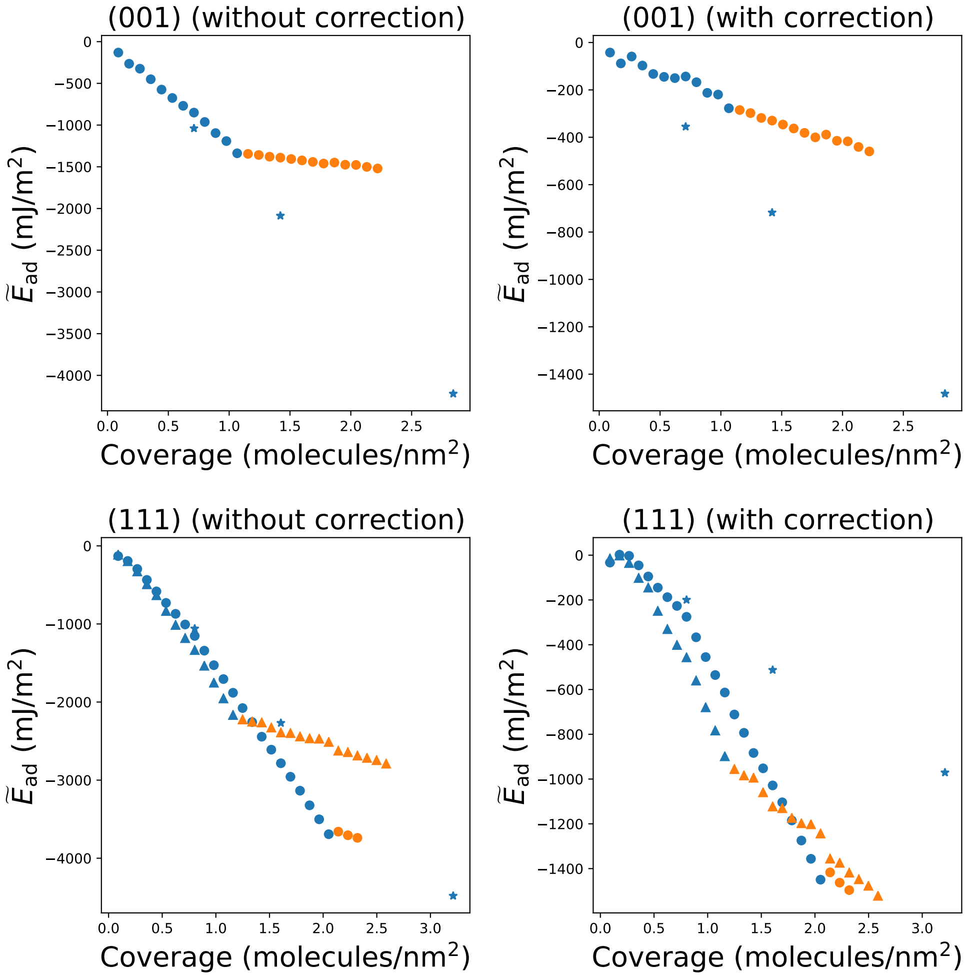

Figure S9: Formation energies at a given coverage of oleate/oleic acid on magnetite surfaces, corrected for the proton transfer energy of a dissociative adsorption. Blue and orange colors represent dissociative adsorption and molecular adsorption, respectively. Triangles refer to a simulation in which further dissociation was not allowed after 1.0 molecules/nm^2. Stars denote the energy of artificially covered surfaces (cf. right panel of Fig. S12 and S13).

netite surface. $\tilde{E}_{\mathrm{ad}}(\lambda)$ is not normalized per molecule and is given in units of $\mathrm{mJ/m^2}$ (surface areas of our slabs comprise $11.25\,\mathrm{nm}^2$ and $11.20\,\mathrm{nm}^2$ for (001) and (111), respectively). $E^{\mathrm{MM}}_{\mathrm{oleic}} = 2.05\,\mathrm{eV}$ and corresponds to the force field energy of an isolated oleic acid molecule in vacuum. Energies without correction for the two surfaces are additionally shown in Fig. S9 for comparison. On closer inspection of the slopes in Fig. S9, it appears that in the case of magnetite (111) we have two different dissociative adsorption regimes that we associate with changes in the adsorption motif, *i.e.*, from quasi-bidentate to chelating (cf. motifs III and IV in Fig. S8b). Comparing the energies with artificially covered surfaces marked with stars in Fig. S9, where oleates were placed by hand on the corresponding adsorption sites, it is likely that, at least in the case of the (001) surface, the final coverage associated with dissociative adsorption is not fully reached in our simulations, which could be explained by the time constraints of the simulations and the high steric hindrance due to the molecules on this surface. Due to the higher mobility of oleate on the (111) surface (*i.e.*, the quasi-bidentate adsorption), this does not seem to be an issue here. However, care should be taken when comparing absolute energy values or different slopes, since the QM correction may be very different due to the different adsorption motifs.

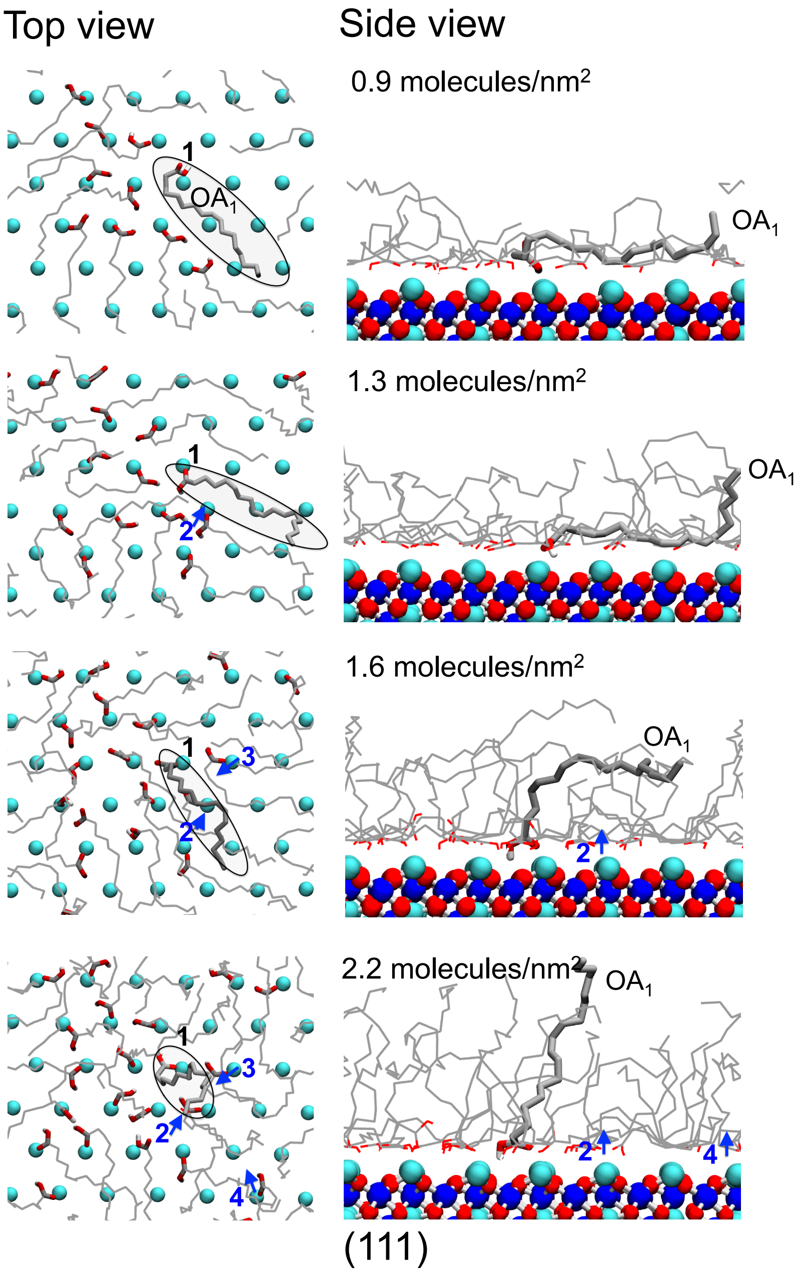

Figure S10: Formation of a monolayer of oleate/oleic acid on magnetite (111). With increasing coverage, restructuring of carboxylates on the surface and a transition from a rather flat to a standing geometry of the oleates are observed. A newly deposited oleic acid OA_1 is indicated by a black ellipse in the top view on the left and highlighted in the side view on the right. More oleic acids, *i.e.*, molecule number 2, 3 and 4, are iteratively deposited afterwards. Each arrow corresponds to the displacement direction of the aliphatic OA_1 chain upon adsorption of more oleic acid. At 2.2 molecules/nm^2 surface coverage the aliphatic chains are oriented upwards. However, the monolayer is still not perfectly ordered. Unlike saturated fatty acids, which are densely packed in nearly crystalline, self-assembled monolayers due to van der Waals forces, we suspect that unsaturated oleic acid molecules are not as readily densely packed due to a double bond in the hydrocarbon chain[13].

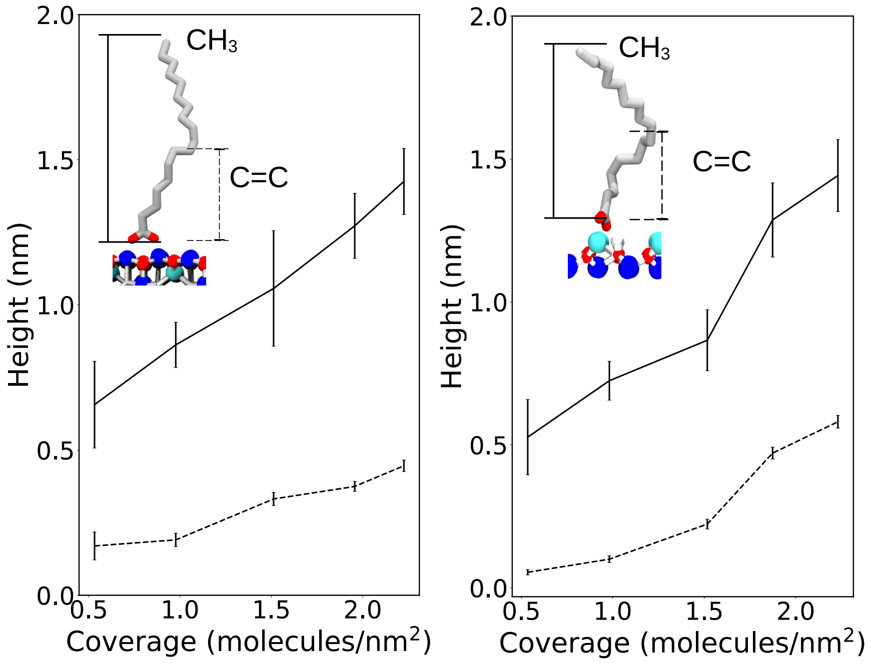

Figure S11: Layer thickness of oleate on the (001)-DBT and Fe_{tet1}-terminated (111) magnetite surfaces at varying coverages. Shown are the height of the CH_3 endgroup and the C=C double bond of the aliphatic tail above the surface. Error bars indicate the height variations of individual oleates.

Table S9: Adsorption geometries of oleic acid on magnetite surfaces at room temperature. Distances and angles are given in Å and degree, respectively.

**Surface**	**Measure**	**Molecular**	**Dissociative**	**Mixed**
(001)	$d_{\mathrm{Fe_{oct}-O_A}}$	2.19±0.15	1.92±0.01	2.01±0.15
	$d_{\mathrm{H_A-O_M}}$	2.53±0.13	n/a	n/a
	$d_{\mathrm{H_A-O_A^*}}$	n/a	n/a	1.57±0.13
	$\angle_{\mathrm{O_A-C-O_H}}$ (I)	123.01±2.91	n/a	122.43±3.13
	$\angle_{\mathrm{O_A-C-O_A}}$ (II)	n/a	117.50±2.95	n/a
	$\angle_{\mathrm{O_A-C-O_A^*}}$	n/a	n/a	118.28±3.22
(111)	$d_{\mathrm{Fe_{tet1}-O_A}}$	2.03±0.01	1.89±0.08	1.98±0.09
	$d_{\mathrm{H_A-O_M}}$	2.51±0.15	n/a	n/a
	$d_{\mathrm{H_A-O_A^*}}$	n/a	n/a	1.55±0.11
	$\angle_{\mathrm{O_A-C-O_H}}$ (I)	122.72±2.91	n/a	122.51±1.99
	$\angle_{\mathrm{O_A-C-O_A^*}}$ (III)	n/a	115.54±3.20	112.12±3.01
	$\angle_{\mathrm{O_A-C-O_A}}$ (IV)	n/a	112.38±3.32	111.95±1.91

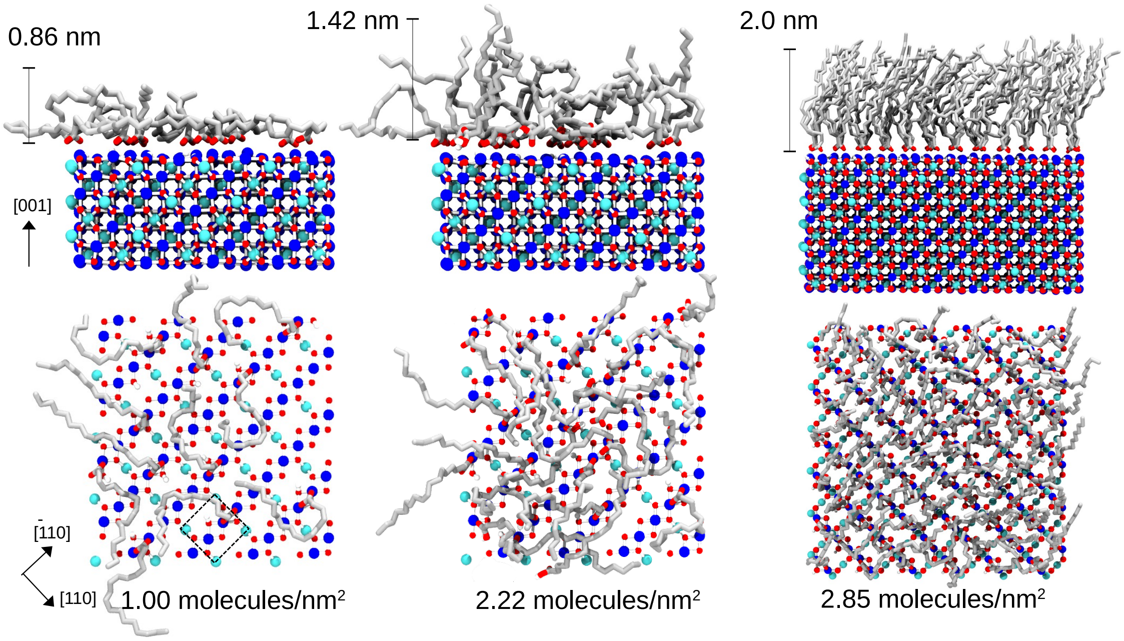

Figure S12: Simulation snapshots for three different oleate coverages on the (001) surface. A coverage of 2.85 molecules/nm^2 was achieved by manually depositing all oleate molecules on the hypothetical adsorption sites for formate (see ref.[8] for details) while the other two lower coverages were obtained by successive random deposition over the surface and dissociative adsorption of oleic acid.

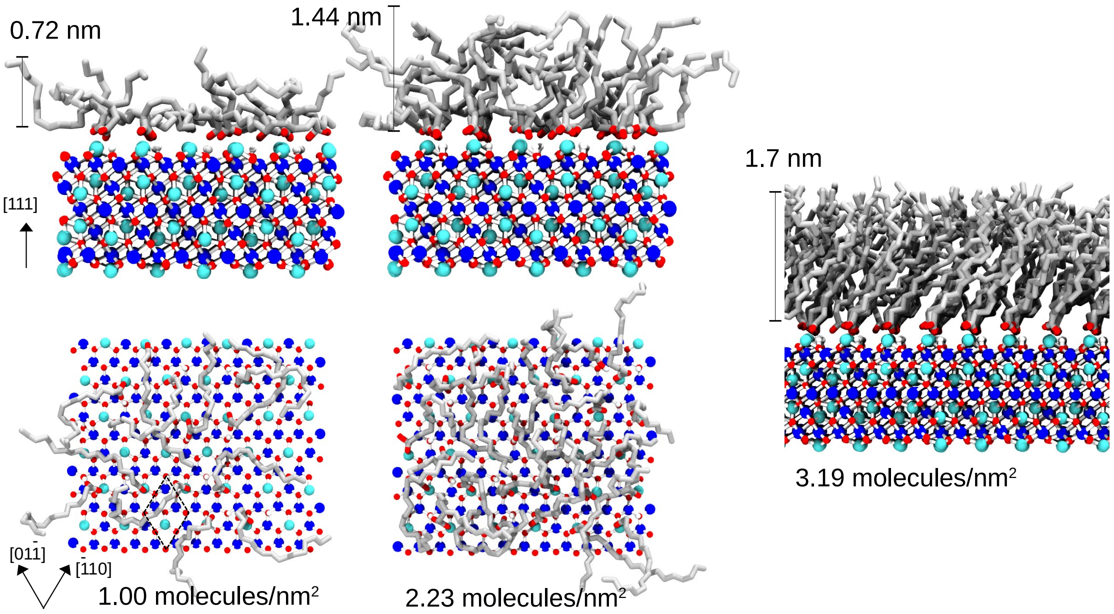

Figure S13: Simulation snapshots for three different oleate coverages on the (111) surface. A coverage of 3.19 molecules/nm^2 was achieved by manually depositing all oleate molecules on almost all hypothetical adsorption sites for formate (see ref. 11 for details) while the other two lower coverages were obtained by successive random deposition over the surface and dissociative adsorption of oleic acid.

Supplementary Movies of oleic acid layer growth

The formation of an oleic acid monolayer on magnetite is visualized in two movies that can be downloaded from the journal's website. Supplementary Movie 1 shows the adsorption on magnetite (001) up to a coverage of 1.01 molecules/nm^2 within a total simulation time of 0.64 ns. The adsorption on magnetite (111) is visualized in Supplementary Movie 2 up to a coverage of 2.05 molecules/nm^2 during a total simulation time of 2.44 ns.

References

(1) Falleiro, R.; Silva, L.; Meirelles, A.; Krähenbühl, M. Vapor pressure data for fatty acids obtained using an adaptation of the DSC technique. *Thermochimica Acta* **2012**, *547*, 6 – 12.

(2) Cappa, C. D.; Lovejoy, E. R.; Ravishankara, A. R. Evaporation Rates and Vapor Pressures of the Even-Numbered C_8-C_{18} Monocarboxylic Acids. *J. Phys. Chem. A* **2008**, *112*, 3959–3964, PMID: 18348549.

(3) Davis, L. E. Handbook of Auger Electron Spectroscopy: A Reference Book of Standard Data for Identification and Interpretation of Auger Electron Spectroscopy Data. *Physical Electronics* **1996**,

(4) Fleet, M. E. The structure of magnetite. *Acta Crystallographica Section B* **1981**, *37*, 917–920.

(5) Rasmussen, M. K.; Foster, A. S.; Hinnemann, B.; Canova, F. F.; Helveg, S.; Meinander, K.; Martin, N. M.; Knudsen, J.; Vlad, A.; Lundgren, E.; Stierle, A.; Besenbacher, F.; Lauritsen, J. V. Stable Cation Inversion at the $MgAl_2O_4$(100) Surface. *Phys. Rev. Lett.* **2011**, *107*, 036102.

(6) Jensen, T. N.; Rasmussen, M. K.; Knudsen, J.; Vlad, A.; Volkov, S.; Lundgren, E.;

Stierle, A.; Lauritsen, J. V. Correlation between stoichiometry and surface structure of the polar $MgAl_2O_4(100)$ surface as a function of annealing temperature. *Phys. Chem. Chem. Phys.* **2015**, *17*, 5795–5804.

(7) Arndt, B.; Bliem, R.; Gamba, O.; Van Der Hoeven, J. E. S.; Noei, H.; Diebold, U.; Parkinson, G. S.; Stierle, A. Atomic structure and stability of magnetite $Fe_3O_4(001)$: An X-ray view. *Surf. Sci.* **2016**, *653*, 76–81.

(8) Arndt, B.; Sellschopp, K.; Creutzburg, M.; Grånäs, E.; Krausert, K.; Vonk, V.; Müller, S.; Noei, H.; Vonbun-Feldbauer, G.; Stierle, A. Carboxylic acid induced near-surface restructuring of a magnetite surface. *Commun. Chem.* **2019**, *2*.

(9) Creutzburg, M.; Sellschopp, K.; Gleißner, R.; Arndt, B.; Vonbun-Feldbauer, G. B.; Vonk, V.; Noei, H.; Stierle, A. Surface Structure of Magnetite (111) under Oxidizing and Reducing Conditions. *J. Phys. Condens. Matter* **2022**, in press, doi: 10.1088/1361-648X/ac4d5a.

(10) Arndt, B.; Creutzburg, M.; Grånäs, E.; Volkov, S.; Krausert, K.; Vlad, A.; Noei, H.; Stierle, A. Water and atomic hydrogen adsorption on magnetite (001). *J. Phys. Chem. C* **2019**, *123*, 26662–26672.

(11) Creutzburg, M.; Sellschopp, K.; Tober, S.; Grånäs, E.; Vonk, V.; Mayr-Schmölzer, W.; Müller, S.; Noei, H.; Vonbun-Feldbauer, G. B.; Stierle, A. Heterogeneous Adsorption and Local Ordering of Formate on a Magnetite Surface. *J. Phys. Chem. Lett.* **2021**, *12*, 3847–3852.

(12) Dietrich, H.; Schmaltz, T.; Halik, M.; Zahn, D. Molecular dynamics simulations of phosphonic acid–aluminum oxide self-organization and their evolution into ordered monolayers. *Physical Chemistry Chemical Physics* **2017**, *19*, 5137–5144.

(13) Siram, K.; Rahman, S. H.; Balakumar, K.; Duganath, N.; Chandrasekar, R.; Hariprasad, R. *Biomedical Applications of Nanoparticles*; Elsevier , 2019; pp 91–115.
