# Supplementary material for: Adsorption of oleic acid on magnetite facets
Source: Commun Chem. 2022 Oct 23;5:134. doi: 10.1038/s42004-022-00741-0 (PMC9814498; doi:10.1038/s42004-022-00741-0)
Supplement: Supplementary file 2 — Description of Additional Supplementary Files [file 42004_2022_741_MOESM2_ESM.docx]

Description of Additional Supplementary Files

**File name:** Supplementary Movie 1

**Description:** The adsorption on magnetite (001) up to a coverage of 1.01 molecules/nm^2^ within a total simulation time of 0.64 ns.

**File name:** Supplementary Movie 2

**Description:** The adsorption on magnetite (111) is visualized in Movie S2 up to a coverage of 2.05 molecules/nm^2^ during a total simulation time of 2.44 ns.
